# Efficacy and mechanisms of andrographolide derivative in acute exacerbations of chronic obstructive pulmonary disease: a meta-analysis, network pharmacology, molecular docking, and *in vitro* validation study

**DOI:** 10.3389/fphar.2026.1915339

**Published:** 2026-09-23

**Authors:** Jiaxiang Li, Xiaowen Wu, Yuning Shen, Jiayang Yao, Zhiyue Yu, Ming Gao, Weihao Gao, Limei Geng

**Affiliations:** 1 Graduate School, Hebei University of Chinese Medicine, Shijiazhuang, Hebei, China; 2 Department of Respiratory and Critical Care Medicine, The First Affiliated Hospital of Hebei University of Chinese Medicine, Shijiazhuang, Hebei, China

**Keywords:** acute exacerbation of COPD, andrographolide derivatives, inflammation, meta-analysis, molecular docking, network pharmacology

## Abstract

**Background:**

Acute exacerbation of chronic obstructive pulmonary disease (AECOPD) contributes substantially to morbidity and mortality. Andrographolide derivative injections (e.g., Xiyanping, Yanhuning, Chuanhuning, Lianbizhi) are widely used adjunctive treatments in China. Their clinical efficacy remains unclear, and we explored the potential mechanisms of the parent compound andrographolide in this study.

**Methods:**

A systematic review and meta-analysis of 32 randomized controlled trials (n = 2,668) comparing andrographolide derivatives plus conventional therapy versus conventional therapy alone in patients with AECOPD was conducted. Pulmonary function (FEV_1_%, FEV_1_, FVC), arterial oxygen tension (PaO_2_), inflammatory markers (CRP, WBC, NEUT%, PCT), and overall clinical response were evaluated. Network pharmacology, molecular docking, and *in vitro* experiments using LPS-stimulated BEAS-2B cells were performed to explore underlying mechanisms.

**Results:**

Adjunctive andrographolide derivatives significantly improved overall clinical response rate [RR = 1.21, 95% CI 1.17–1.26, P < 0.00001], FEV_1_% [MD = 6.48, 95% CI 3.52–9.45], FEV_1_ [MD = 0.30 L, 95% CI 0.20–0.40], FVC [MD = 0.28 L, 95% CI 0.10–0.46], and PaO_2_ [MD = 6.56 mmHg, 95% CI 4.68–8.45]. Inflammatory markers were also reduced: CRP [MD = −4.72 mg/L, 95% CI -6.83 to –2.61], WBC [MD = −1.30 ×10^9^/L, 95% CI −1.79 to −0.80], NEUT% [MD = −4.36 percentage points, 95% CI -6.18 to −2.54], PCT [MD = −0.17 ng/mL, 95% CI -0.23 to −0.10] (all P < 0.0001). Network pharmacology identified 39 overlapping targets, highlighting TNF, IL-1β, NLRP3, and CASP1 as core targets. Molecular docking suggested energetically favorable predicted interactions of andrographolide with NLRP3, caspase-1, IL-1β, and IL-18. *In vitro*, andrographolide enhanced BEAS-2B cell viability, reduced LDH release, and suppressed NLRP3, caspase-1, IL-1β, and TNF-α expression.

**Conclusion:**

Andrographolide derivative injections as adjunctive therapy may improve clinical response, pulmonary function, oxygenation, and inflammatory status in patients with AECOPD. Mechanistic experiments using the parent compound andrographolide suggest that these therapeutic effects are potentially mediated by the NLRP3/caspase-1/IL-1β inflammatory axis. Nevertheless, these findings should be interpreted cautiously given the exclusively Chinese single-center RCTs, substantial heterogeneity in some outcomes, and lack of direct mechanistic data on individual derivatives.

## Graphical Abstract

**Figure:**
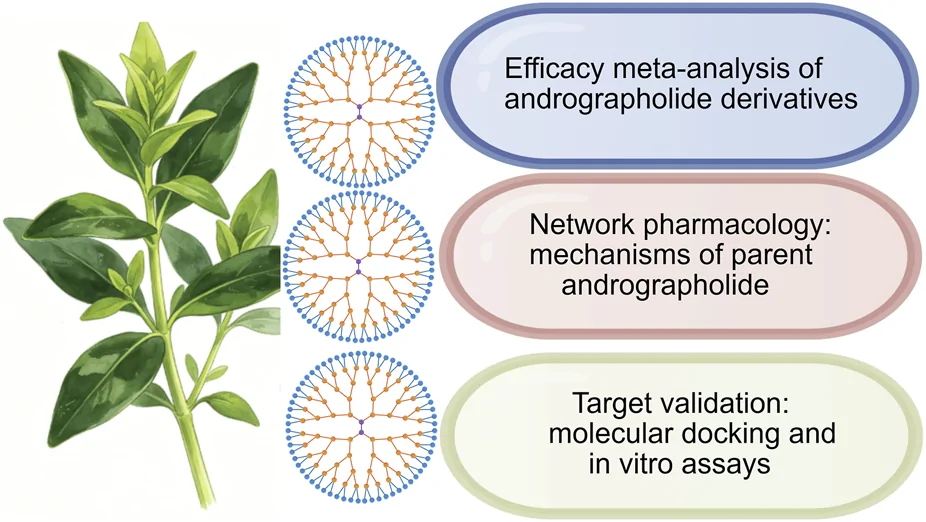

## 1 Introduction

Chronic obstructive pulmonary disease (COPD) is a prevalent chronic airway disorder that is closely associated with prolonged exposure to tobacco smoke, either active or passive, as well as environmental pollutants. These risk factors ultimately lead to sustained oxidative stress and persistent inflammatory responses within the airway (Wiegman et al., 2020; Xu et al., 2024; Ambrocio-Ortiz et al., 2021; Barnes, 2016). As a major global public health concern, acute exacerbations of COPD (AECOPD) are responsible for a marked decline in patients’ quality of life, an accelerated deterioration of lung function, increased mortality risk, and a substantial economic burden on healthcare systems. The cumulative global direct medical expenditure attributable to COPD is projected to reach USD 24.35 trillion by 2050 (Boers et al., 2025). Data from the REAL study indicate that AECOPD remains a major clinical challenge in China (Yang et al., 2023). Current management focuses on rapidly controlling inflammation, relieving airflow obstruction, and improving symptoms, but important therapeutic limitations remain (Schroedl and Kalhan, 2012; Tu et al., 2025; Brennan et al., 2022). Therefore, there is an urgent unmet clinical need to develop safer and more effective therapeutic strategies for AECOPD. Traditional Chinese medicine (TCM) demonstrates distinctive advantages and well-established clinical value in the prevention and management of chronic airway diseases (Bai et al., 2023). Accumulating evidence has demonstrated that TCM intervention can significantly enhance the therapeutic efficacy of AECOPD management, reduce the adverse effects of conventional Western medications, and improve the long-term prognosis of affected patients (Guo et al., 2025; Li et al., 2024; Zhu et al., 2024).

Within the theoretical framework of TCM, COPD is classified as “lung distension” and “panting syndrome.” Acute exacerbations are generally considered to result from an overabundance of pathogenic factors and are clinically manifested by aggravated cough, increased sputum production, and wheezing (Lenferink et al., 2017). The principal pathogenesis is often described as internal accumulation of phlegm-heat with impaired dispersing and descending functions of lung qi (Cao et al., 2023). Accordingly, heat-clearing, phlegm-resolving, and cough-relieving therapies are frequently used to alleviate inflammation and symptoms (Smith and Calverley, 2004). Andrographolide (AD) is a diterpenoid lactone compound isolated from Andrographis paniculata (Burm. f.) Nees and is considered one of its major bioactive constituents (Zeng et al., 2024). Andrographis paniculata is recorded in authoritative Chinese materia medica references, including Zhong Yao Zhi and the National Compendium of Chinese Herbal Medicine, for conditions associated with febrile illness, sore throat, parotitis, and damp-heat diarrhea (Chinese Academy of Medical Sciences Institute of Materia Medica, 1961; Wang, 2014). AD, which serves as the major bioactive compound underlying the pharmacological effects of Andrographis paniculata, retains the fundamental medicinal properties of this herbal medicine (Gonde et al., 2024). As a single bioactive component belonging to the diterpenoid lactone class, AD possesses a unique chemical structure. Its core pharmacological activities are centered on anti-inflammation, antioxidation, immunomodulation, and airway mucosal protection. It can specifically alleviate airway inflammatory responses and inhibit oxidative stress injury, which provides a solid pharmacological rationale for its use in the treatment of chronic airway disorders (Raman et al., 2022).

AD exhibits potent anti-inflammatory and antiviral effects: it suppresses inflammatory mediators, reduces airway mucosal edema and exudation, modulates systemic immune function, and enhances airway defense capacity, thereby exerting a synergistic anti-inflammatory and lung-protective effect (Sandborn et al., 2013; Jadhav and Karuppayil, 2021; Jiang et al., 2021; Dai et al., 2019). Compared with conventional Western medications, AD offers the advantages of fewer adverse effects, lower toxicity, and a lower propensity for drug resistance. It addresses the unmet need of modern patients with COPD for safer, milder, and more targeted therapies, and is particularly suitable for elderly patients with acute airway diseases who have poor treatment tolerance (Dai et al., 2019).

Several andrographolide derivative injections are used clinically in China, including Xiyanping, Yanhuning, Chuanhuning, and Lianbizhi injections (Zhang et al., 2021). These preparations are produced by structural modification of andrographolide or dehydroandrographolide, and Xiyanping is the most widely used. Chen et al., (2022) reported that Xiyanping injection reduced inflammation and improved pulmonary function in patients with COPD. Although Yanhuning, Chuanhuning, and Lianbizhi are prescribed less frequently, they possess similar anti-inflammatory and anti-infective activities and have also been applied in the management of respiratory diseases.

Accordingly, this investigation was undertaken to comprehensively assess both the therapeutic efficacy and potential mechanisms of andrographolide derivatives for AECOPD by integrating meta-analysis, network pharmacology, molecular docking, and *in vitro* experimental validation. To the best of our knowledge, this is the first study to synthesize clinical evidence of all four major andrographolide derivative injections for AECOPD and to validate their potential anti-inflammatory mechanisms at the airway epithelial cell level. In addition, molecular docking together with *in vitro* experiments was performed to further validate the potential interactions between AD, the major bioactive precursor compound of these derivatives, and critical inflammatory targets and signaling pathways. This evidence-integrated mechanistic framework not only strengthens the mechanistic rationale supporting the adjunctive application of andrographolide derivatives in AECOPD but also establishes a scientific foundation for their rational clinical use and future pharmaceutical development in airway inflammatory diseases.

## 2 Materials and methods

### 2.1 Protocol registration and reporting

This systematic review and meta-analysis was registered with PROSPERO (CRD420261282003) and was conducted and reported in accordance with the Preferred Reporting Items for Systematic Reviews and Meta-Analyses (PRISMA) 2020 statement (Page et al., 2021).

### 2.2 Search strategy

Seven electronic databases were searched from inception to 31 March 2026: PubMed, Web of Science, the Cochrane Library, Elsevier ScienceDirect, China National Knowledge Infrastructure (CNKI), Wanfang Data, and the VIP Database. Reference lists of relevant studies and reviews were also screened. The search was performed using a combination of medical subject headings and free-text terms for “chronic obstructive pulmonary disease”, “acute exacerbation”, and “andrographolide”. The complete search strategies are provided in Supplementary Table 1.

All evaluated injections are derived from AD. Structural and chemical differences among AD, Xiyanping, Yanhuning, Chuanhuning, and Lianbizhi are summarized in Table 1.

**TABLE 1:** Chemical differences among andrographolide, Xiyanping, Yanhuning, Chuanhuning, and Lianbizhi.

English name	Parent compound	Chemical modification	Manufacturers
Andrographolide	Andrographolide (diterpenoid lactone)	Natural compound, no modification	—
Chuanhuning	Dehydroandrographolide	Succinylation + salt formation	Zhenbaodao Pharma, Harbin Sanjing Pharma, Xi’an Lijun Pharma
Yanhuning	Dehydroandrographolide	Succinylation + salt formation	Chongqing Yaoyou Pharma, Humanwell Pharma, Shuanglu Pharma
Xiyanping	Andrographolide	Sulfonation	Jiangxi Qingfeng pharmaceutical co., Ltd
Lianbizhi	Andrographolide	Sodium bisulfite addition	Shineway Pharma, Wuxi Jiyu Shanhe, Jiangsu Jiuxu

Additionally, the reference lists of relevant original studies and systematic reviews were manually screened to identify potentially eligible studies that were not captured by electronic searches. Title and abstract screening and full-text assessment were independently performed by two reviewers (Jiayang Yao and Xiaowen Wu) to minimize selection bias. Any discrepancies were resolved through consultation with a third reviewer (Zhiyue Yu).

### 2.3 Eligibility criteria

Studies were eligible if they met all of the following criteria:

1. Randomized controlled trial;
2. Participants diagnosed with COPD according to the Global Initiative for Chronic Obstructive Lung Disease (GOLD) criteria and presenting with acute worsening of cough, sputum production or wheezing;
3. The intervention group received an andrographolide derivative injection in addition to conventional therapy, whereas the control group received the same conventional therapy alone;
4. Studies reported at least one of the following outcome measures: overall clinical response rate, forced expiratory volume in 1 s as a percentage of the predicted value (FEV_1_%), forced expiratory volume in 1 s (FEV_1_), forced vital capacity (FVC), C-reactive protein (CRP), white blood cell count (WBC), neutrophil percentage (NEUT%), procalcitonin (PCT), or arterial oxygen tension (PaO_2_).

Studies meeting any of the following conditions were excluded:

1. Non-randomized studies, retrospective analyses, animal experiments, reviews, or other non-RCT designs;
2. Participants had major non-airway comorbidities likely to confound treatment effects, such as active malignancy, decompensated heart failure, or severe hepatic or renal dysfunction;
3. The intervention group received additional Chinese herbal medicines or proprietary Chinese medicines other than the specified andrographolide derivative;
4. Outcome data were incomplete or internally inconsistent and could not be clarified after attempts to contact the authors;
5. Duplicate publication.

### 2.4 Data extraction

Two reviewers (Ming Gao and Xiaowen Wu) independently extracted data, and disagreements were resolved by discussion among the authors. Extracted information included publication details (title, first author, and year), study design and treatment duration, participant characteristics (sample size, mean age, and sex distribution), intervention and comparator regimens, and outcome data.

Extracted outcomes included:

1. Clinical outcome: overall clinical response rate.
2. Oxygenation outcome: PaO_2_.
3. Pulmonary function outcomes: FEV_1_%, FEV_1_, and FVC.
4. Inflammatory outcomes: CRP, WBC, NEUT% and PCT.

When data were unclear, incomplete, or inconsistent, the corresponding authors were contacted for clarification. The included products were approved injectable formulations manufactured by licensed pharmaceutical manufacturers. Product names, manufacturers, and reported dosages were extracted whenever available (Xu et al., 2021). Detailed information is provided in Table 1.

### 2.5 Risk of bias and certainty of evidence

References were organized and managed with Zotero 7.0.27. Risk-of-bias assessment was performed independently by two reviewers (Xiaowen Wu and Zhiyue Yu) according to the Cochrane Risk tool. The evaluation covered random sequence generation, allocation concealment, blinding of participants and personnel, blinding of outcome assessment, incomplete outcome data, selective reporting, and other potential sources of bias. Each domain was classified as having a low, high, or unclear risk of bias. The certainty of evidence for the primary outcomes was evaluated using the Grading of Recommendations Assessment, Development and Evaluation (GRADE) framework (Guyatt et al., 2008).

### 2.6 Statistical analysis

Quantitative analyses were conducted using Review Manager 5.4 and Stata 17.0. For dichotomous variables, effect estimates were expressed as risk ratios (RRs) or odds ratios (ORs), whereas continuous variables were pooled as mean differences (MDs) or standardized mean differences (SMDs), together with their corresponding 95% confidence intervals (CIs). Statistical heterogeneity among studies was evaluated using Cochran’s Q test and the I^2^ statistic. A fixed-effect model was adopted when the heterogeneity assessment indicated P ≥ 0.05 and I^2^ < 50%; otherwise, a random-effects model was employed. Statistical significance was defined at α = 0.05 (Higgins et al., 2003). Leave-one-out sensitivity analysis was undertaken for outcomes exhibiting substantial heterogeneity. Prespecified subgroup analyses were subsequently performed based on injection type, age, and treatment duration. Publication bias and small-study effects were assessed using funnel plots and Egger’s regression test when an adequate number of studies were available. In cases where obvious funnel-plot asymmetry was detected, the trim-and-fill approach was applied as an exploratory correction method.

### 2.7 Identification of AD -related targets

The chemical structure of AD was obtained from the PubChem Database (https://pubchem.ncbi.nlm.nih.gov/). Putative drug targets were identified using the HERB Database, PharmMapper Database (https://www.lilab-ecust.cn/pharmmapper/), and SwissTargetPrediction Database (http://www.swisstargetprediction.ch/). The union of targets from the three databases was retained to ensure capture of all potential targets (Janani et al., 2022). Target gene names were standardized using the UniProt Database (https://www.uniprot.org/) to generate a corresponding standardized target list (UniProt Consortium, 2025).

### 2.8 Identification of AECOPD-related targets

AECOPD-related targets were retrieved from GeneCards and OMIM using the species restriction “*Homo sapiens*.” After merging, deduplication, and UniProt standardization, the disease-target dataset was established (Shan et al., 2022).

### 2.9 Network construction and enrichment analysis

Overlapping targets between AD-related targets and AECOPD-associated gene targets were identified using Venn diagram analysis in the Venny 2.1.0 platform (https://bioinfogp.cnb.csic.es/tools/venny/), which represent the potential therapeutic targets of AD against AECOPD. These common targets were then uploaded to the STRING Database (https://string-db.org/) to construct a protein-protein interaction (PPI) network and elucidate the interactions among them (Szklarczyk et al., 2021). Based on the overlapping targets identified by Venn analysis, a topological network of drug-active ingredient-target-disease was constructed using Cytoscape software (version 3.9.0). Subsequently, the common targets were imported into the Metascape Database (https://metascape.org/gp/index.html) for further enrichment analysis to annotate their functions according to the biological processes they participate in (Zhou et al., 2019). The top 5 most significantly enriched Gene Ontology (GO) terms in the cellular component (CC), biological process (BP), and molecular function (MF) categories, as well as the top 10 most important Kyoto Encyclopedia of Genes and Genomes (KEGG) pathways, were selected according to adjusted P value (FDR) in descending order of -log10 (adjusted P value).

### 2.10 Molecular docking

AD was defined as the ligand, and core target proteins were selected as receptors. Crystal structures were downloaded from the RCSB Protein Data Bank (PDB). Ligand and receptor structures were pretreated using AutoDock Tools by hydrogenation, charge calculation, and water removal. The grid box was set to cover the active pocket of each protein. AutoDock 4.2 was used for molecular docking, and PyMOL 2.5 was applied for visualization of binding conformations. Binding energy was calculated for each ligand–receptor complex using AutoDock 4.2. The predicted docking energies were used for comparative interpretation, with lower (more negative) values indicating more energetically favorable predicted interactions (Morris et al., 2009).

### 2.11 Cell culture and experimental groups

Human bronchial epithelial BEAS-2B cells (Sunncell, SNL-203, Wuhan, China) were cultured in high-glucose DMEM containing 10% fetal bovine serum and 1% penicillin–streptomycin at 37 °C in a humidified incubator with 5% CO_2_. Cells in the logarithmic growth phase were selected for all subsequent experiments.

BEAS-2B cells were plated in culture dishes and exposed to LPS (100 ng/mL) for 24 h to establish an inflammatory epithelial model (Tao et al., 2026; Yuan et al., 2018). Cells in the control group were left untreated, whereas those in the model group were exposed to LPS alone. The treatment groups received AD (Solarbio, IA0360, Beijing, China) in combination with LPS. Following the designated intervention period, both cells and culture supernatants were harvested for subsequent analyses. Routine *mycoplasma* testing was performed throughout the study. Each experiment included three independent biological replicates and three technical replicates for each sample.

### 2.12 Cell viability assay

Cell viability was assessed using the Cell Counting Kit-8 (CCK-8) assay, a classical method for evaluating cellular activity. Cells were seeded into 96-well plates and treated according to the experimental groups. Subsequently, CCK-8 reagent (Solarbio, CA1210, Beijing, China) was added to each well, and the plates were incubated at 37 °C in the dark. The absorbance at 450 nm was measured using a microplate reader, and cell viability was calculated accordingly. Cells were seeded at a density of 5 × 10^3^ cells per well. The incubation time for CCK-8 reagent was 2 h.

### 2.13 LDH release assay

LDH levels were determined using an LDH Assay Kit (Solarbio, BC0685, Beijing, China). Culture supernatants collected from each experimental group were subjected to analysis in accordance with the manufacturer’s protocol. Absorbance values were recorded at 450 nm, and LDH concentrations were quantified using a standard calibration curve to evaluate cellular LDH release.

### 2.14 Cytokine measurement

IL-1β (Solarbio, SEKH-0002, Beijing, China) and TNF-α (Solarbio, SEKH-0047, Beijing, China) in culture supernatants were quantified according to the manufacturers’ ELISA protocols.

### 2.15 Western blotting

Protein expression of NLRP3 (Solarbio, K004108P, Beijing, China) and caspase-1 (Solarbio, K011502M, Beijing, China) was assessed by Western blotting. Equal amounts of protein were loaded onto SDS-PAGE gels for electrophoresis, followed by transmembrane. After membrane blocking with 5% skim milk, primary antibody incubation was carried out overnight at 4 °C, followed by incubation with the appropriate secondary antibodies at room temperature for 1 h. GAPDH was employed as the internal control protein. Band intensities were visualized and analyzed using ImageJ software.

### 2.16 Immunofluorescence

NLRP3 expression and localization were examined by immunofluorescence. Cells were fixed, permeabilized, incubated with the primary antibody and a fluorescent secondary antibody, mounted, and imaged by fluorescence microscopy.

### 2.17 *In vitro* statistical analysis

Results obtained from the *in vitro* studies are presented as mean ± SD. Data normality was assessed using the Shapiro–Wilk test. Comparisons among groups were performed using one-way ANOVA with Tukey’s *post hoc* multiple-comparison test. A two-sided P value less than 0.05 was considered indicative of statistical significance. Statistical analyses were carried out using GraphPad Prism 9.0 software.

## 3 Results

### 3.1 Clinical evidence from the meta-analysis

#### 3.1.1 Study selection

Initially, the literature search yielded 172 relevant records. After removing duplicates, 95 records remained. Subsequently, 42 studies were selected for full-text assessment following initial screening of titles and abstracts. Based on the predefined eligibility criteria, ten articles were excluded after full-text assessment: four were non-randomized studies, and six enrolled ineligible populations, including patients with stable COPD or severe complications. Finally, 32 studies were included in the meta-analysis after full-text review. The literature search and screening process is shown in Figure 1. Thirty-two RCTs (Chen and Liu, 2013; Fu et al., 2013; Chang, 2015; Ding et al., 2007; Han and Li, 2012; Han and Zhang, 2015; Hao et al., 2019; Hou et al., 2016; Hu and Xiao, 2016; Hu, 2014; Liang et al., 2012; Liu et al., 2016; Ma and Li, 2008; Qian and Song, 2013; Tao and Xiao, 2011; Wang et al., 2018; Wu, 2015; Wu et al., 2016; Xu, 2025; Yao et al., 2020; Ceng, 2018; Zhang et al., 2017; Zhang H. et al., 2014; Zhang, 2011; Zhang and Liu, 2017; Zhang and Chen, 2011; Zhang X. et al., 2014; Zhang and Zhang, 2013; Zhao and Yu, 2022; Zhao et al., 2019; Zhang and Pang, 2018; Peng et al., 2014) involving 2,668 participants were included: 1,342 in the andrographolide derivative group and 1,326 in the control group. All studies were conducted in mainland China. Treatment duration ranged from 5 to 21 days. Conventional therapy included oxygen therapy, antibiotics, nebulized bronchodilators, glucocorticoids, and other symptomatic treatments. The intervention groups received an andrographolide derivative injection in addition to the same conventional therapy. Study characteristics are summarized in Table 2.

**FIGURE 1:**
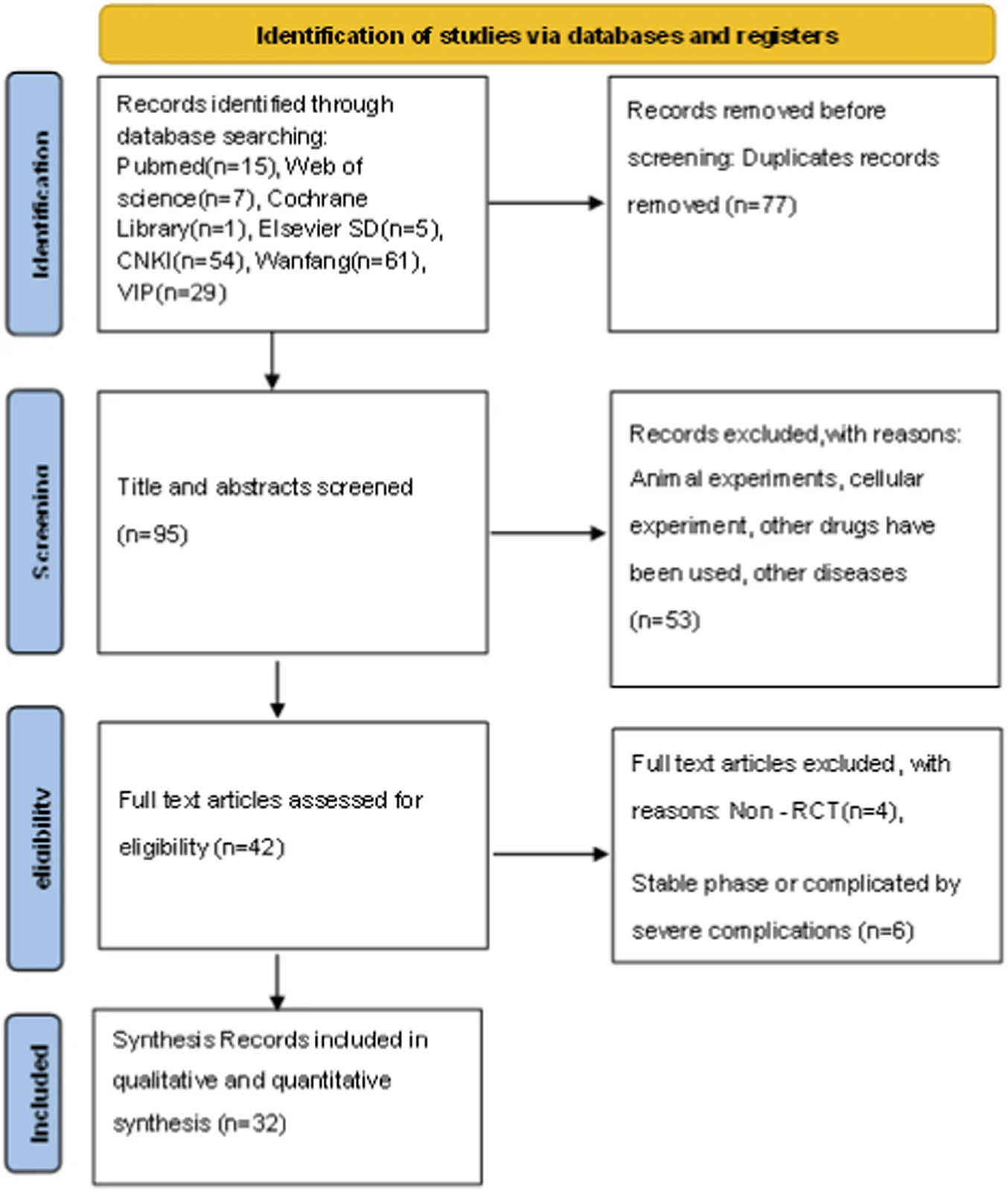
PRISMA flow diagram of study selection.

**TABLE 2:** Characteristics of the included randomized controlled trials.

Study, year	Injection	Intervention	Sample size (T/C)	Duration (days)	Male (T/C)	Age, T	Age, C	Outcomes
Ceng et al., 2018	Xiyanping	CT + Xiyanping IV	59/59	14	37/34	67.7 ± 11.6	69.3 ± 11.4	a
Chang et al., 2015	Xiyanping	CT + Xiyanping IV	44/44	10	22/24	66	66	a, g, h
Chen and Li 2013	Andrographolide sulfonates	CT + andrographolide sulfonates, nebulized	48/48	7	27/24	60.4 ± 16.3	58.3 ± 15.7	b, c, f
Ding et al., 2007	Xiyanping	CT + Xiyanping IV	27/25	14–21	10/15	51.4	52.1	a
Fu et al., 2013	Chuanhuning	CT + Chuanhuning IV	30/30	14	17/14	67.4 ± 9.7	70.2 ± 10.5	b, c
Han and Li 2012	Xiyanping	CT + Xiyanping IV	23/27	14	15/17	61	61	a, c, f
Han and Zhang 2015	Xiyanping	CT + Xiyanping IV	62/60	14	37/33	56	61	b, c, d
Hao et al., 2019	Yanhuning	CT + Yanhuning IV	61/61	14	37/35	68.44 ± 5.36	67.46 ± 5.93	a, b, d, i
Hou et al., 2016	Xiyanping	CT + Xiyanping IV	30/30	10	—	—	—	f, g, h, i
Hu and Xiao 2016	Xiyanping	CT + Xiyanping IV	50/50	5	28/26	70.7 ± 11.3	70.9 ± 10.8	b, c
Hu, 2014	Xiyanping	CT + Xiyanping IV	30/30	10–14	18/16	61.7	62.2	a, g, h
Liang et al., 2012	Xiyanping	CT + Xiyanping IV	15/15	14	—	59	59	a, b, c, d
Liu et al., 2016	Xiyanping	CT + Xiyanping IV	30/30	—	16/17	69.00 ± 5.73	68.10 ± 5.94	a, b, c, f
Ma and Li 2008	Xiyanping	CT + Xiyanping IV	26/22	—	15/13	74	75	a, b, c, d
Qian and Song 2013	Xiyanping	CT + Xiyanping IV	30/30	10	17/19	58	63	a, b, c, g, h
Tao and Ziao 2011	Yanhuning	CT + Yanhuning IV	58/52	10	—	63.03 ± 4.60	63.03 ± 4.60	a, b, d, e
Wang et al., 2018	Xiyanping	CT + Xiyanping IV	50/50	14	32/30	65.35 ± 3.10	64.92 ± 3.45	a
Wu, 2015	Xiyanping	CT + Xiyanping IV	40/40	14	38/34	65.73 ± 3.54	65.11 ± 3.34	a
Wu et al., 2016	Xiyanping	CT + Xiyanping IV	45/48	7	—	—	—	a
Xu, 2025	Xiyanping	CT + Xiyanping IV	30/30	7	17/18	58.25 ± 4.17	57.68 ± 4.12	a, f, i
Yao et al., 2020	Chuanhuning	CT + Chuanhuning IV	50/50	14	28/30	56.62 ± 7.27	56.85 ± 7.45	a, d, e
Zhang et al., 2017	Chuanhuning	CT + Chuanhuning IV	48/43	14	29/25	59.13 ± 7.22	58.61 ± 6.84	a, f, i
Zhang et al., 2014b	Xiyanping	CT + Xiyanping IV	40/40	10	31/33	62.8 ± 3.5	63.1 ± 3.8	a, b, c, f, g, h
Zhang et al., 2011	Xiyanping	CT + Xiyanping IV	32/30	10	20/17	66	64	a
Zhang et al., 2017	Xiyanping	CT + Xiyanping IV	45/45	10	21/20	45.89 ± 4.69	46.21 ± 4.59	a, d, e
Zhang et al., 2011	Xiyanping	CT + Xiyanping IV	40/40	10	22/24	68.3 ± 5.5	66.9 ± 7.4	a, f, g, h
Zhang et al., 2014a	Xiyanping	CT + Xiyanping IV	46/46	14	30/32	67.8 ± 7.4	67.6 ± 7.2	a, d, e, f
Zhang et al., 2013	Xiyanping	CT + Xiyanping IV	50/50	10	—	—	—	b, c, d
Zhao et al., 2022	Xiyanping	CT + Xiyanping IV	50/50	14	29/30	50.79 ± 1.29	51.39 ± 1.37	a, b, e
Zhao et al., 2019	Xiyanping	CT + Xiyanping IV	49/47	7	26/25	66.9 ± 6.2	68.1 ± 5.4	a, g
Peng et al., 2014	Xiyanping	CT + Xiyanping IV	40/40	10	22/21	69.13 ± 5.28	68.36 ± 5.67	a
Zhang et al., 2018	Xiyanping	CT + Xiyanping IV	64/46	14	42/31	50–65	50–68	a, d, f, g, i

Abbreviations: CT, conventional therapy; IV, intravenous infusion; T, treatment group; C, control group. Outcomes: a, overall clinical response rate; b, PaO_2_; c, FEV1%; d, FEV1; e, FVC; f, CRP; g, WBC; h, NEUT%; i, PCT.

#### 3.1.2 Risk of bias and certainty of evidence

Among the 32 RCTs, 10 reported random-number-table methods and were rated as low risk for random sequence generation, whereas 22 did not describe the randomization method and were rated as unclear risk. None of the studies described allocation concealment. Peng et al. (2014) reported double blinding; the remaining studies did not report blinding and were rated as unclear risk in the relevant domains. All studies reported complete outcome data, and no attrition-related concerns were identified. One study (Chen et al., 2013) used nebulized administration, which was considered a potential source of additional bias. The risk-of-bias graph is shown in Figure 2 and the study-level summary in Supplementary Figure S1. According to GRADE, four outcomes were rated as moderate-certainty evidence and four as low-certainty evidence. GRADE downgrades were mainly attributed to high risk of bias, substantial heterogeneity, and potential publication bias (detailed assessment in Supplementary Table 2).

**FIGURE 2:**
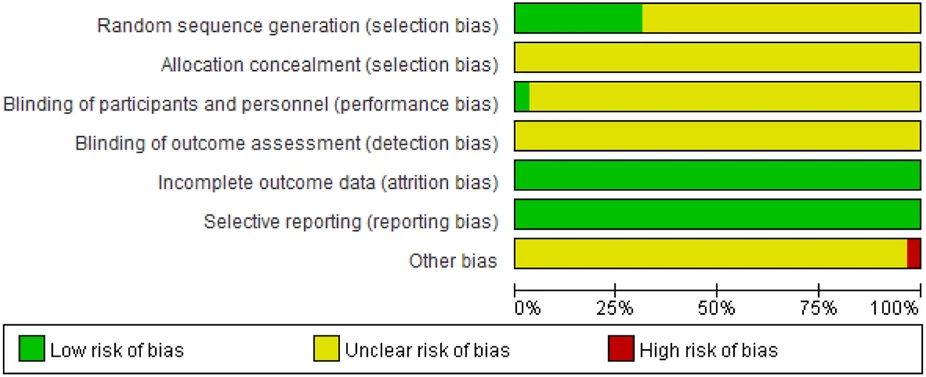
Risk of bias graph.

#### 3.1.3 Overall clinical response rate

The overall clinical response rate was a key composite endpoint of clinical signs and symptoms. Given the negligible heterogeneity (I^2^ = 0%, P = 0.93), a fixed-effect model was applied. The results showed that intervention with andrographolide derivative injections resulted in a statistically significant improvement in the response rate compared with the control group [RR = 1.21, 95% CI (1.17, 1.26), P < 0.00001], as shown in Figure 3. Sensitivity analysis demonstrated the robustness of the pooled result. Since 26 studies were included, funnel plot and Egger’s regression test were further performed, which indicated the presence of small-study effects (P < 0.001). However, after adjustment using the trim-and-fill method (with the number of studies imputed to 37), the recalculated adjusted pooled effect size remained statistically significant (RR = 1.162, 95% CI [1.125, 1.201]; P < 0.001), confirming the robustness of the primary conclusion, as shown in Supplementary Figure 2. Subgroup analyses were conducted based on injection type, age, and treatment duration. No significant subgroup differences were observed for injection type (P = 0.78), age (P = 0.41), or treatment duration (P = 0.28). Detailed results are provided in Supplementary Table 3.

**FIGURE 3:**
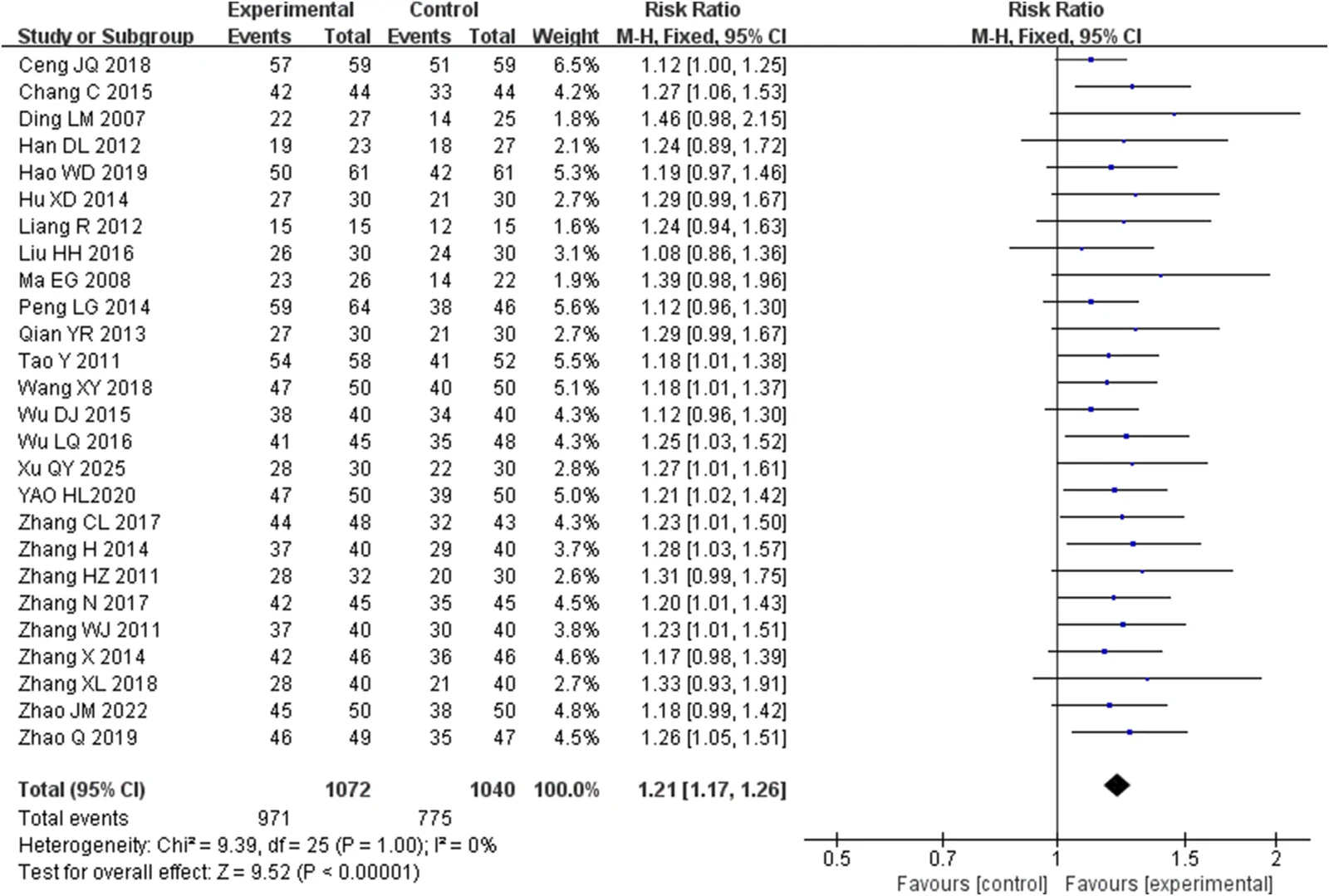
Forest plot of the overall clinical response rate.

#### 3.1.4 Arterial oxygen tension

13 studies reported PaO_2_. Heterogeneity was substantial (I^2^ = 76%, P < 0.00001), and a random-effects model was used. The intervention improved PaO_2_ compared with conventional therapy alone (MD = 6.56, 95% CI 4.68–8.45; P < 0.00001; Figure 4). Exclusion of Hu et al. reduced I^2^ from 76% to 32%, without materially changing the pooled effect (MD = 5.61, 95% CI 4.40–6.82; P < 0.00001). Since 13 studies were included, funnel plot and Egger’s regression test were further performed, which indicated no significant small-study effects (P = 0.116 > 0.05), as shown in Supplementary Figure 3. Subgroup differences were not significant for age (P = 0.93) or treatment duration (P = 0.86), but differed by injection type (P = 0.03), suggesting that formulation may partly explain heterogeneity (Supplementary Table 3).

**FIGURE 4:**
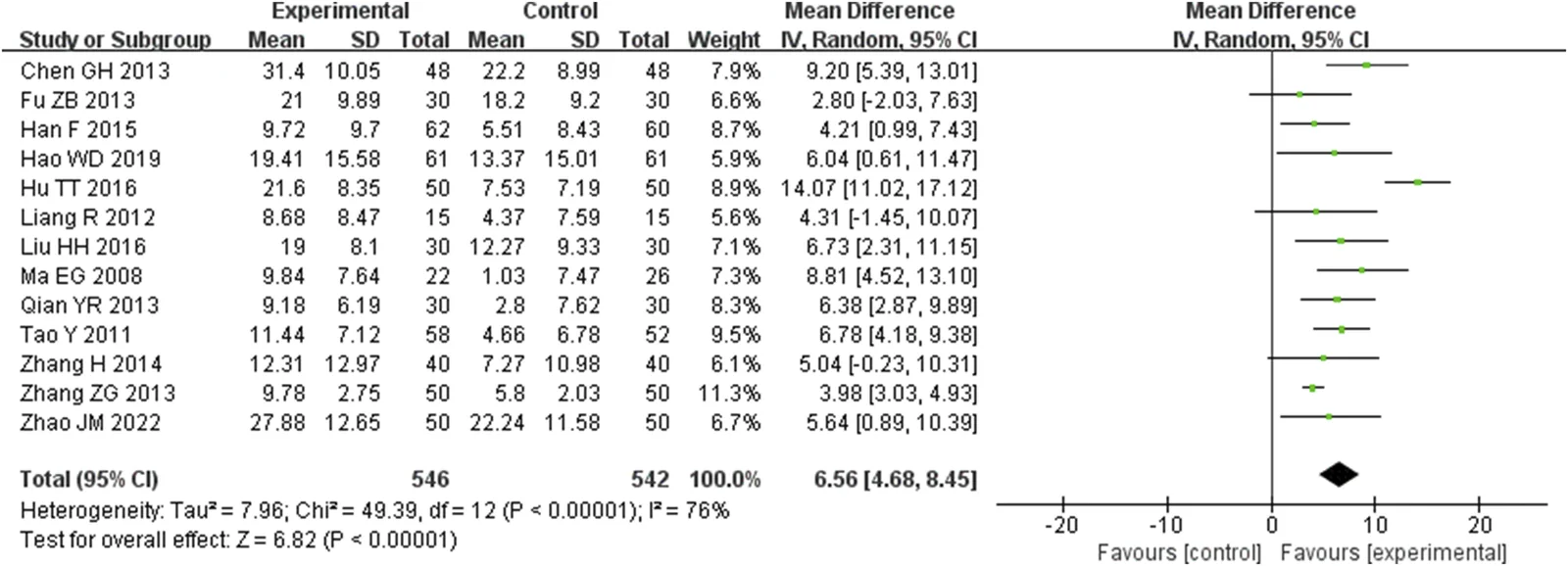
Forest plot of arterial oxygen tension (PaO_2_).

#### 3.1.5 Pulmonary function

Pulmonary function parameters including FEV1%, FEV1 and FVC serve as core indicators for evaluating patients’ respiratory function.

##### 3.1.5.1 FEV_1_%

A total of 11 studies reported FEV_1_% outcomes. Owing to substantial heterogeneity (I^2^ = 93%, P < 0.00001), a random-effects model was adopted. Pooled results demonstrated a statistically remarkable elevation in FEV_1_% following adjunctive andrographolide derivative injection compared with the control group [MD = 6.48, 95% CI (3.52, 9.45), P < 0.00001], as presented in Figure 5. Sensitivity analysis verified the stability of the pooled estimate. Funnel plot and Egger’s regression test were carried out across the 11 included studies, revealing no evidence of small-study effects (P = 0.116 > 0.05; Supplementary Figure 4). Subgroup analyses stratified by injection formulation, patient age and treatment duration detected no statistically significant intergroup differences (P = 0.63, 0.94 and 0.68, respectively). Full details are listed in Supplementary Table 3.

**FIGURE 5:**
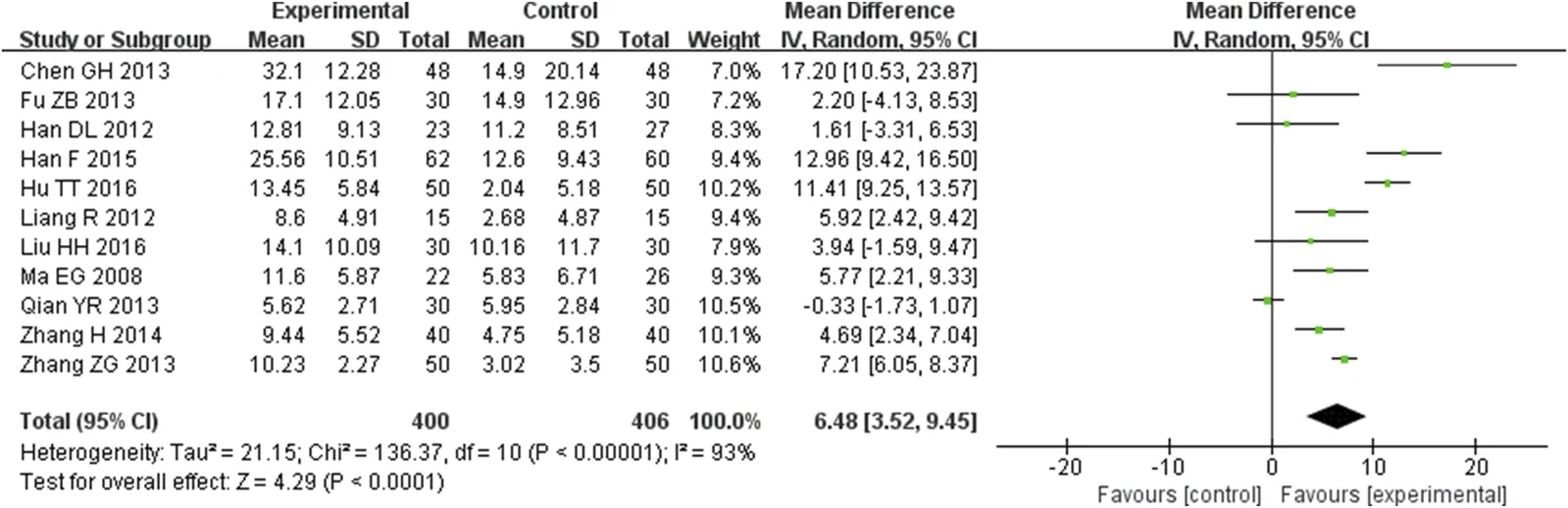
Forest plot of forced expiratory volume in 1 s as a percentage of the predicted value (FEV_1_%).

##### 3.1.5.2 FEV1

Ten studies reported FEV_1_. Heterogeneity was substantial (I^2^ = 88%, P < 0.00001), and a random-effects model was used. The intervention improved FEV_1_ (MD = 0.30, 95% CI 0.20–0.40; P < 0.00001; Figure 6). Sensitivity analysis supported the robustness of the result. Funnel plot and Egger’s regression test were performed based on the 10 included studies, which indicated an absence of small-study effects (P = 0.418), as shown in Supplementary Figure 5. Subgroup differences were not significant for injection type (P = 0.68) or treatment duration (P = 0.33), but differed by age (P = 0.02).

**FIGURE 6:**
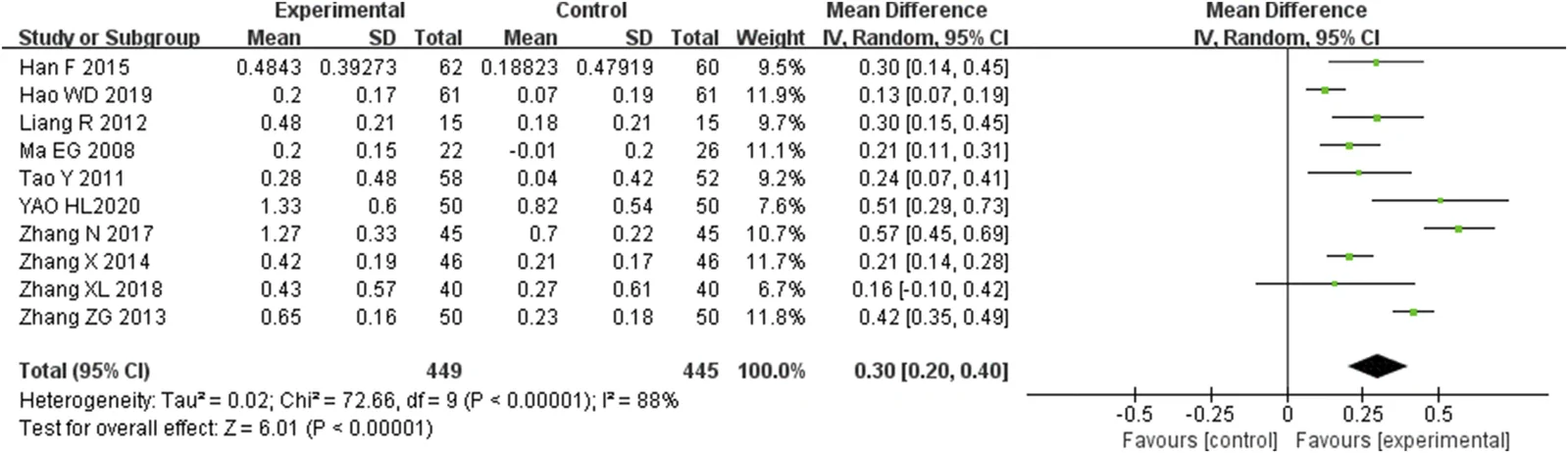
Forest plot of forced expiratory volume in 1 s (FEV_1_).

##### 3.1.5.3 FVC

Five studies reported FVC. Heterogeneity was substantial (I^2^ = 83%, P < 0.00001), and a random-effects model was used. The intervention improved FVC (MD = 0.28, 95% CI 0.10–0.46; P = 0.003; Figure 7). Sensitivity analysis supported the robustness of the result. No significant subgroup differences were observed by age (P = 0.84) or treatment duration (P = 0.68).

**FIGURE 7:**
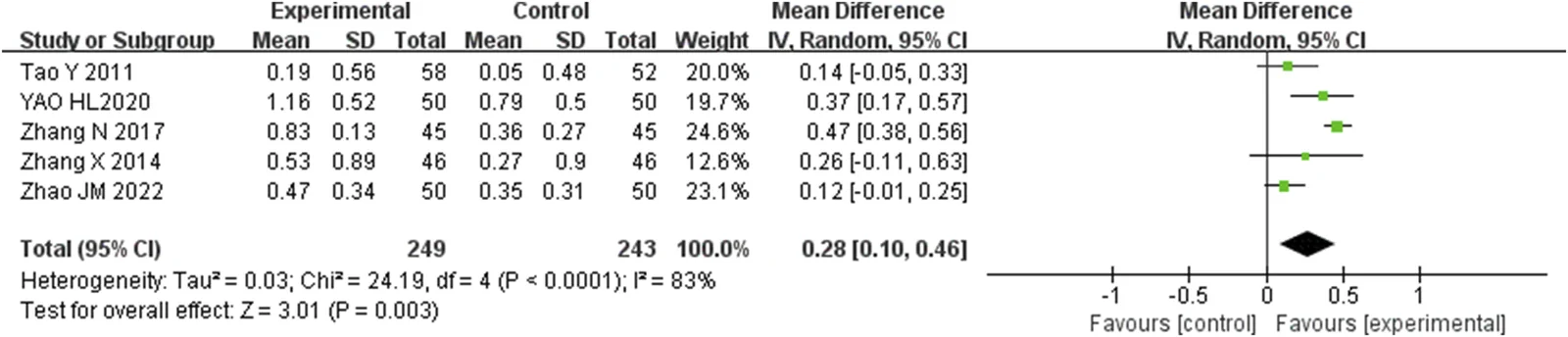
Forest plot of forced vital capacity (FVC).

#### 3.1.6 Inflammatory markers

##### 3.1.6.1 C-reactive protein

Ten studies reported CRP. Heterogeneity was moderate (I^2^ = 49%, P = 0.04), and a random-effects model was used. The pooled estimate of the improvement favored adjunctive andrographolide derivative injections (MD = −4.72 mg/L, 95% CI −6.83 to −2.61; Figure 8). Sensitivity analysis indicated a stable result. Funnel plot and Egger’s regression test were performed based on the 10 included studies, which indicated no significant small-study effects (P = 0.110 > 0.05), as shown in Supplementary Figure 6. Subgroup analyses stratified by age and treatment duration revealed statistically significant intergroup differences (P = 0.02 for age, P = 0.04 for treatment duration), suggesting that both age and treatment duration may be potential sources of heterogeneity in CRP outcomes. Notably, subgroup analysis indicated that the subgroup of patients aged >65 years and the subgroup with treatment duration >14 days exhibited a more pronounced improvement in CRP levels. Detailed results are provided in Supplementary Table 3.

**FIGURE 8:**
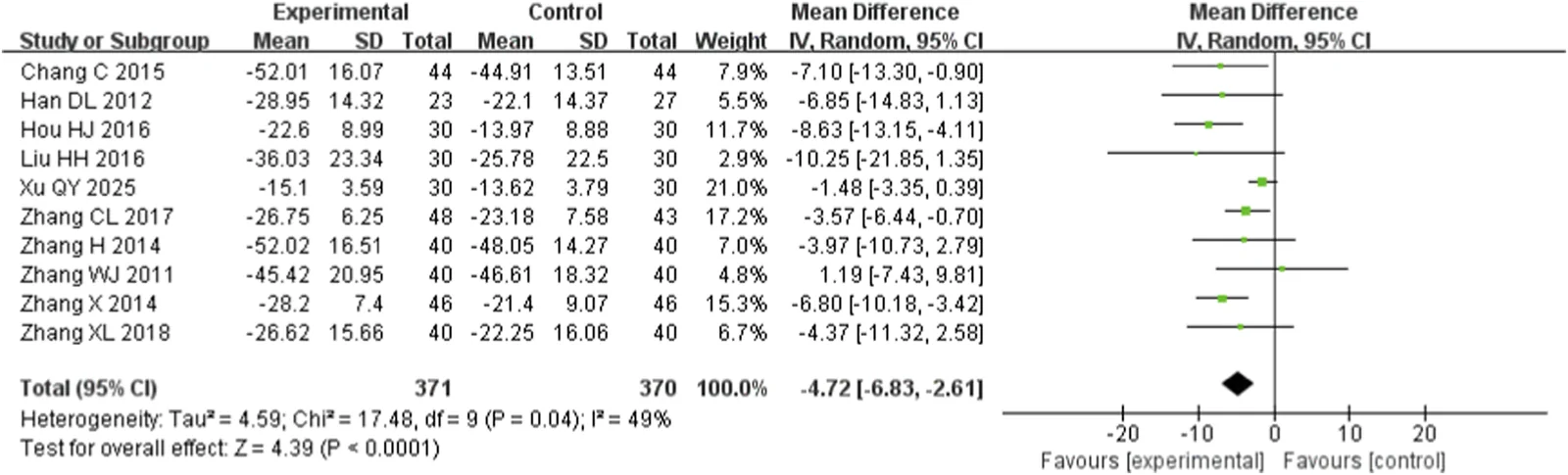
Forest plot of C-reactive protein (CRP).

##### 3.1.6.2 White blood cell count

Eight studies reported WBC. Heterogeneity was low (I^2^ = 7%, P = 0.38), and a fixed-effect model was used. The pooled estimate of the improvement favored adjunctive andrographolide derivative injections (MD = −1.30 ×10^9^/L, 95% CI −1.70 to −0.80; Figure 9). Sensitivity analysis indicated a robust result. No significant subgroup difference was observed by age (P = 0.17). Detailed results are provided in Supplementary Table 3.

**FIGURE 9:**
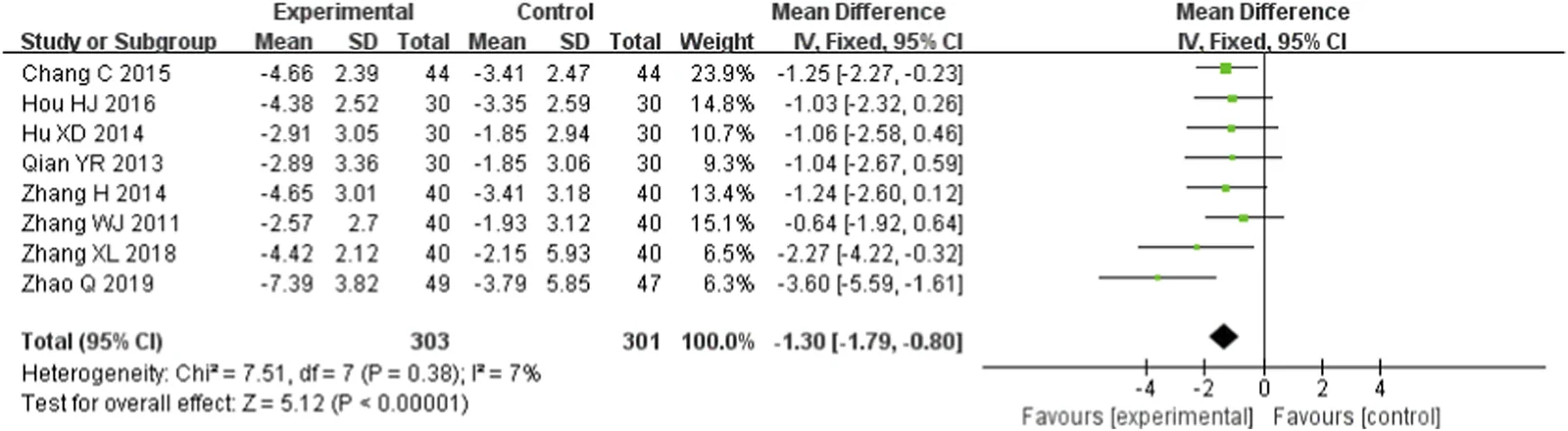
Forest plot of white blood cell count (WBC).

##### 3.1.6.3 NEUT%

Six studies reported NEUT%. Heterogeneity was moderate to substantial (I^2^ = 64%, P = 0.02), and a random-effects model was used. The pooled estimate of the improvement favored adjunctive andrographolide derivative injections (MD = −4.36 percentage points, 95% CI -6.18 to −2.54; Figure 10). Sensitivity analysis supported the robustness of the result. No significant subgroup difference was observed by age (P = 0.52). Detailed results are provided in Supplementary Table 3.

**FIGURE 10:**
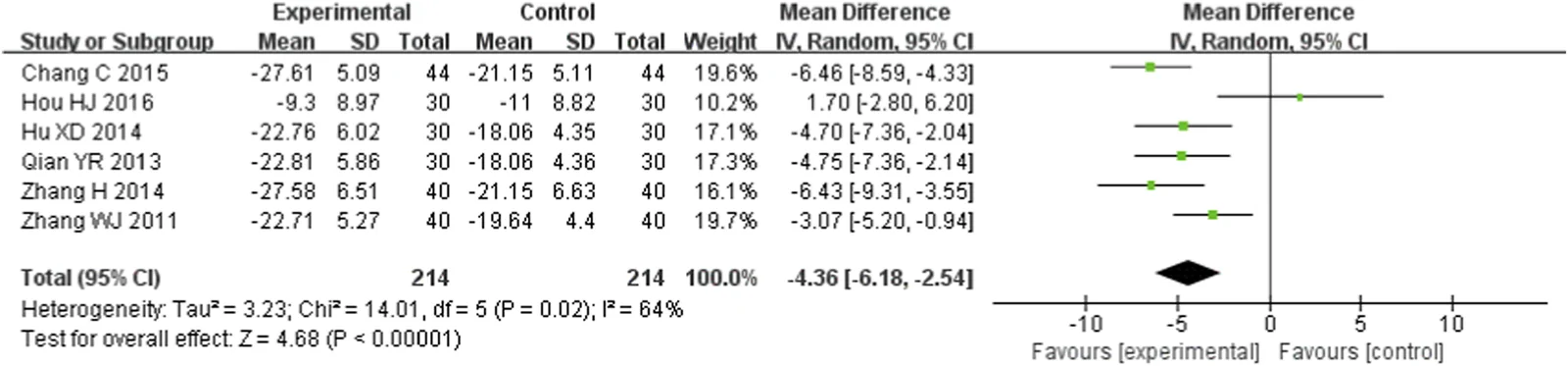
Forest plot of neutrophil percentage (NEUT%).

##### 3.1.6.4 Procalcitonin

Five studies reported PCT. Heterogeneity was negligible (I^2^ = 0%, P = 0.59), and a fixed-effect model was used. The pooled estimate of the improvement favored adjunctive andrographolide derivative injections (MD = −0.17 ng/mL, 95% CI -0.23 to −0.10; Figure 11). Sensitivity analysis indicated a robust result. No significant subgroup differences were observed by injection type (P = 0.67) or treatment duration (P = 0.55). Detailed results are provided in Supplementary Table 3.

**FIGURE 11:**
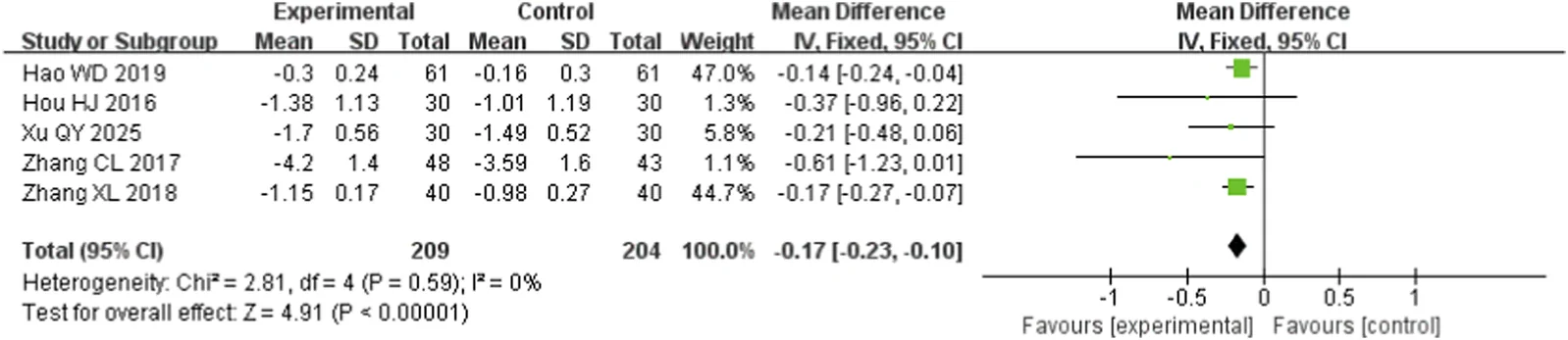
Forest plot of procalcitonin (PCT).

### 3.2 Network pharmacology

#### 3.2.1 Target screening and compound–disease network

Ninety-three putative AD targets and 4,234 AECOPD-related targets were identified after deduplication. Venn analysis identified 39 overlapping genes (Figure 12A). A compound–AECOPD–target network containing 95 nodes and 152 edges was generated in Cytoscape 3.9.0 (Figure 12B).

**FIGURE 12:**
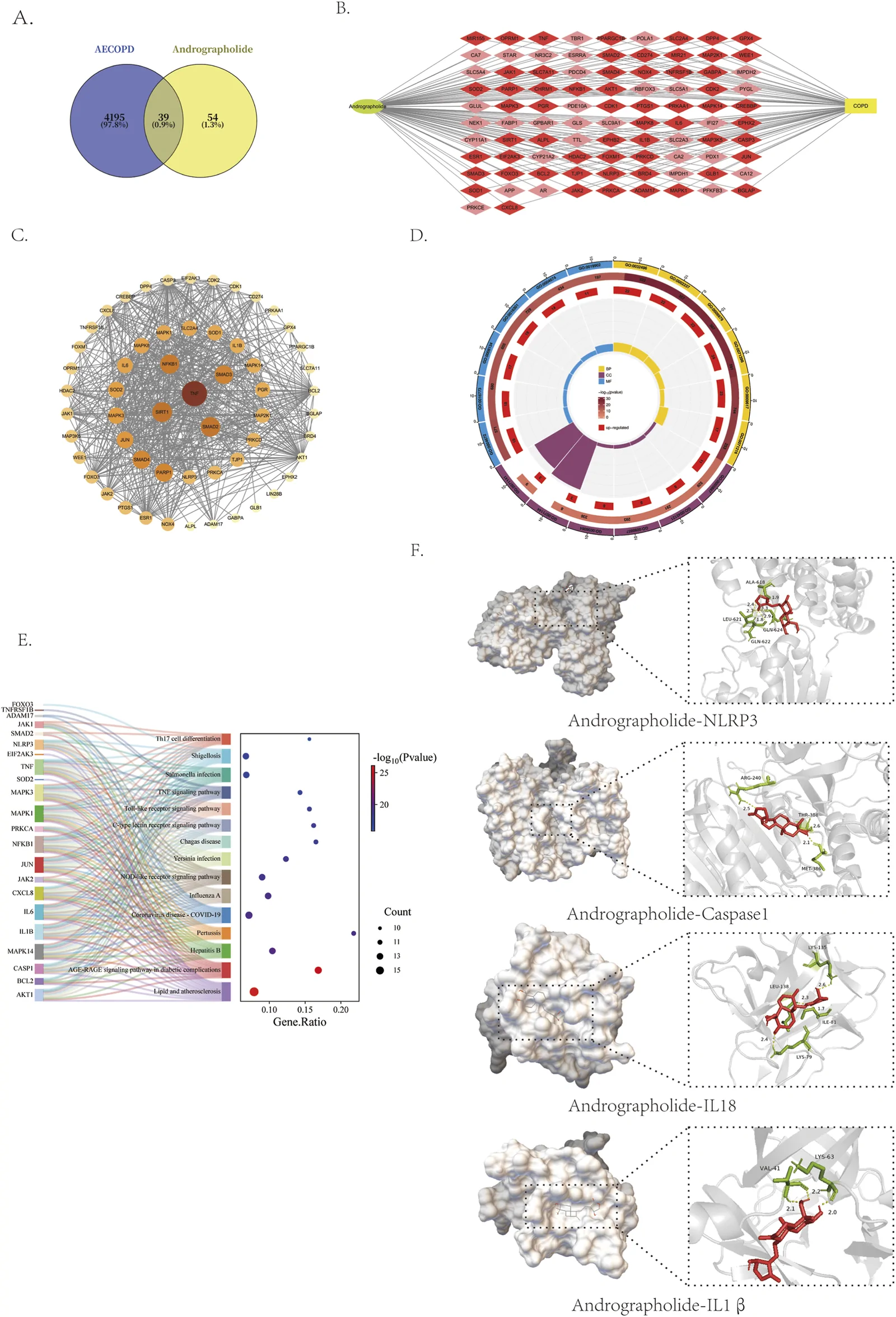
Network pharmacology results. **(A)** Venn diagram showing 39 overlapping targets. **(B)** Compound–AECOPD–target network. **(C)** Protein–protein interaction (PPI) network. **(D)** GO enrichment analysis. **(E)** KEGG pathway enrichment analysis. **(F)** Three-dimensional binding conformations from molecular docking.

#### 3.2.2 Protein–protein interaction network

The STRING-derived PPI network contained 39 nodes and 652 edges (Figure 12C). Core targets included AKT1, TNF, IL1B, CASP1, and NLRP3.

#### 3.2.3 GO and KEGG enrichment analyses

GO enrichment analysis showed significant enrichment in protein kinase activity and growth factor receptor binding within the molecular function category; responses to lipopolysaccharide and bacterium-derived molecules within the biological process category; and membrane rafts and membrane microdomains within the cellular component category (Figure 12D).

KEGG analysis identified several pathways relevant to AECOPD, with the NOD-like receptor signaling pathway among the most prominent (Figure 12E).

### 3.3 Molecular docking

Based on the PPI and KEGG results, IL-18, IL-1β, NLRP3, and caspase-1 were selected for docking with AD. The reported Protein Data Bank identifiers included 3E4C for caspase-1, 7ALV for NLRP3, 1T4Q for IL-1β, and 3F62 for IL-18. The chemical structure of AD was obtained from PubChem.

The binding energies of AD with IL-18, IL-1β, NLRP3, and caspase-1 were −6.16, −6.93, −7.90, and −7.61 kcal/mol, respectively. These results suggested favorable predicted interactions between AD and the four target proteins, especially NLRP3 and caspase-1. Binding conformations and interaction sites are shown in Figure 12F.

### 3.4 *In vitro* validation

Previous studies suggested that AD can modulate NLRP3 and caspase-1 in smoke-exposed mice (Zhang et al., 2026), but its direct effects on airway epithelial cells remained unclear. BEAS-2B cells were therefore stimulated with LPS to establish an LPS-stimulated BEAS-2B epithelial injury model. CCK-8 assays showed that AD at 0, 1, 5,and 10 μmol/L did not reduce cell viability (Figures 13A,B), and 5 and 10 μmol/L were selected for subsequent experiments. LDH release assay serves as a critical indicator for evaluating the degree of epithelial cell injury (Xu et al., 2026). The results showed that LPS increased LDH release into the culture medium, whereas AD significantly reduced LDH release (both P < 0.01; Figure 13C). The NLRP3 inflammasome acts as a central hub linking innate immunity and adaptive immunity (Feng et al., 2022). LPS also upregulated the protein expression levels of NLRP3 and caspase-1 (both P < 0.001), whereas AD downregulated the expression of both proteins (both P < 0.05; Figures 13D–F). ELISA showed that LPS increased IL-1β and TNF-α levels in culture supernatants (both P < 0.001), whereas AD reduced both cytokines (both P < 0.01; Figures 13G,H). Immunofluorescence further demonstrated increased NLRP3 fluorescence intensity after LPS stimulation and attenuation after AD treatment (Figures 13I,J), consistent with the Western blot results. These findings indicate that AD attenuated LPS-induced inflammatory injury in airway epithelial cells, potentially through modulation of the NLRP3/caspase-1 axis.

**FIGURE 13:**
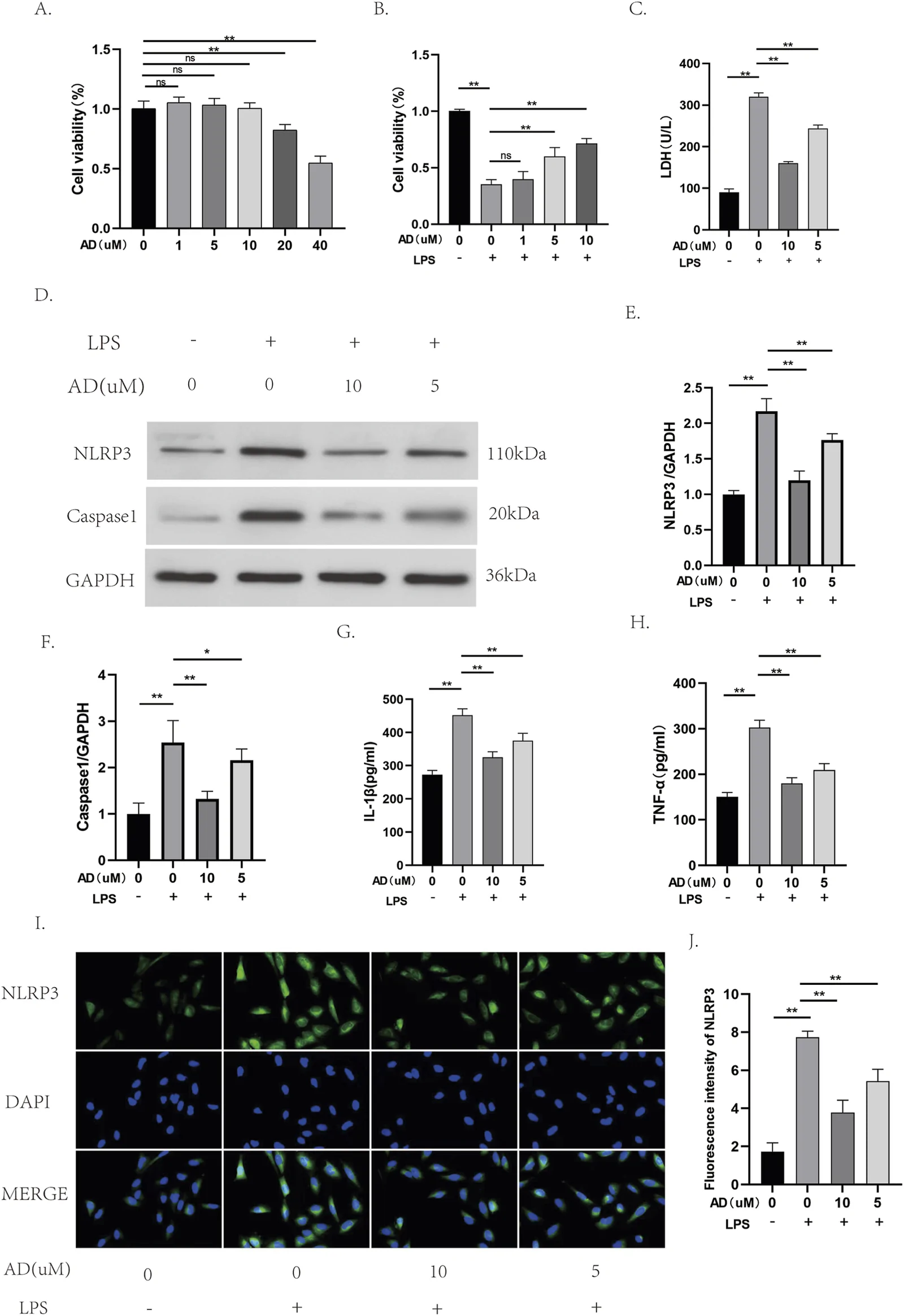
*In vitro* validation in LPS-stimulated BEAS-2B cells. **(A)** Cell viability assessed by CCK-8 assay after treatment with AD at 0, 1, 5, 10, 20, and 40 μmol/L. **(B)** Effects of AD at 1, 5, and 10 μmol/L on cell viability in LPS-stimulated BEAS-2B cells. **(C)** LDH release. **(D–F)** Western blotting of NLRP3 and caspase-1 protein expression. **(G)** IL-1β concentration (ELISA). **(H)** TNF-α concentration (ELISA). **(I,J)** Immunofluorescence of NLRP3. Data are expressed as mean ± SD (n = 3 independent experiments). *P < 0.05; **P < 0.01.

## 4 Discussion

The integration of syndrome differentiation–based traditional Chinese medicine with guideline-recommended conventional treatment has become an important strategy for the comprehensive management of COPD (Li L. et al., 2025). AECOPD represents a major acute episode during the progression of COPD. It is characterized by a marked short-term worsening of core symptoms, including cough, sputum production, and wheezing, and is often accompanied by intensified airway inflammation and an acute decline in pulmonary function. AECOPD is therefore a major contributor to disease progression, rehospitalization, and mortality in patients with COPD (Nisip et al., 2025; Yehia et al., 2024). Several RCTs have shown that heat-clearing and detoxifying Chinese patent medicines, such as Tanreqing and Qingke Pingchuan granules, can alleviate acute symptoms, improve arterial blood gas parameters and pulmonary function, and reduce systemic inflammation in patients with AECOPD (Wang et al., 2026; Li et al., 2026). Although these medicines are widely used for AECOPD in China, no unified guideline currently specifies the optimal treatment duration or dosage form. Their precise mechanisms in regulating airway inflammation and improving airflow limitation also remain incompletely understood. Moreover, the specific effects and clinical efficacy of traditional Chinese medicine interventions for AECOPD have not yet been adequately supported by large-sample, multicenter systematic reviews and meta-analyses.

Andrographis paniculata is a traditional Chinese medicinal herb known for its pronounced anti-inflammatory activity and has attracted considerable attention in the management of inflammatory disease exacerbations (Yu et al., 2024a; Li Z. et al., 2025). Consequently, AD, the major bioactive component of this herb, possesses substantial research significance. In the current study, an integrated approach combining meta-analysis, network pharmacology, molecular docking, and *in vitro* experimental validation was employed to evaluate the therapeutic efficacy and explore the potential pharmacological mechanisms of AD and its derivatives in AECOPD treatment.

Our meta-analysis provided preliminary evidence regarding the clinical effectiveness of andrographolide-derivative injections for AECOPD. The pooled findings suggested that andrographolide derivatives may improve pulmonary function, attenuate inflammation, and relieve clinical symptoms. In particular, the total effective rate was higher when andrographolide-derivative injections were added to conventional treatment than when conventional treatment was administered alone. The intervention group also showed greater improvement in the PaO_2_. After the study by Hu et al. was excluded in the sensitivity analysis, heterogeneity was substantially reduced, while the direction and statistical stability of the pooled result remained unchanged. One possible explanation is that the study by Hu et al. used an observation period of only 5 days, whereas detectable changes in PaO_2_ may require a longer follow-up period. In addition, multiple inflammation-related indicators were significantly reduced, supporting a potentially strong anti-inflammatory effect of andrographolide derivatives.

However, the robustness of the pooled estimates varied across outcomes. Substantial between-study heterogeneity was observed for PaO_2_ (I^2^ = 76%), FEV_1_% (I^2^ = 93%), FEV_1_ (I^2^ = 88%), and FVC (I^2^ = 83%). Although sensitivity analyses did not materially alter the direction of the pooled effects, the magnitude of these estimates should be interpreted cautiously. Subgroup analyses suggested that injection formulation may partly contribute to heterogeneity in PaO_2_ and that age may be associated with heterogeneity in FEV_1_; however, the prespecified subgroup factors did not adequately account for the overall between-study variability. Other potential contributors include differences in derivative formulation and dosage, treatment duration, baseline disease severity, concomitant conventional therapies, and methods used to assess pulmonary function and oxygenation. Therefore, these pooled estimates should be regarded as average effects across clinically and methodologically heterogeneous studies rather than precise effect sizes applicable to all patients with AECOPD. Safety outcomes, including adverse events and hepatic and renal function, could not be quantitatively synthesized because most studies reported them descriptively and used inconsistent definitions and assessment standards. Overall, andrographolide derivative injections combined with conventional treatment appeared to have an acceptable safety profile, and most studies reported no serious adverse events. Mild events, including diarrhea, nausea, vomiting, oral ulceration, and dry mouth, were occasionally reported; however, the safety evidence was insufficient because adverse-event reporting was incomplete, inconsistent, and generally not prespecified. Future trials should report adverse events in greater detail and according to standardized criteria to permit a more comprehensive evaluation of the safety of andrographolide derivative injections combined with conventional treatment. Taken together, the available evidence indicates that this combination may be superior to conventional treatment alone in improving overall therapeutic response, pulmonary function, and inflammatory status in patients with AECOPD, although the certainty of evidence ranged from low to moderate across outcomes.

To further examine whether age, dosage form, and treatment duration modified the intervention effect, subgroup analyses were performed. For PaO_2_, a statistically significant between-subgroup difference was observed only for dosage form, whereas differences according to age and treatment duration were not significant. For FEV_1_, a significant between-subgroup difference was detected only for age, with no significant differences for dosage form or treatment duration. CRP levels differed significantly across both age subgroups and treatment-duration subgroups, suggesting that age and treatment duration may influence changes in CRP. By contrast, no statistically significant between-subgroup differences were observed for FEV_1_%, FVC, WBC, NEUT%, or PCT. These findings suggest outcome-specific effect modification: dosage form may be particularly relevant to PaO_2_, age may be associated with changes in FEV_1_ and CRP, and treatment duration may primarily affect CRP. None of these factors appeared to materially modify the effects on FEV_1_%, FVC, WBC, NEUT%, or PCT. These results may inform the development of individualized treatment strategies. Nevertheless, although age, dosage form, and treatment duration influenced selected outcomes, stratification by these factors did not substantially reduce between-study heterogeneity, indicating that other confounding factors may also have contributed.

Several methodological limitations should be taken into account when evaluating the results of this study. First, substantial heterogeneity was observed for several outcomes, particularly PaO_2_ and pulmonary-function parameters. Although subgroup and sensitivity analyses were performed, the observed heterogeneity was not fully explained. In addition to injection formulation, participant age, and treatment duration, differences in dosage, baseline disease severity, concomitant conventional therapies, and outcome measurement methods may also have contributed. Consequently, the magnitude of the pooled effects for these outcomes should be interpreted with caution. Second, all included RCTs were single-center studies conducted in mainland China, generally with moderate sample sizes and limited representation in English-language databases. These characteristics may restrict the generalizability of the findings. Third, most studies did not evaluate the long-term safety of andrographolide derivative injections, and methods and criteria for reporting adverse events were inconsistent, precluding quantitative synthesis. Accordingly, future studies are warranted to include large-scale, multicenter RCTs characterized by prolonged follow-up and standardized study protocols with enhanced methodological transparency. Moreover, additional research is needed to assess the therapeutic efficacy and safety of these interventions among patients presenting with concomitant airway complications, which may help clarify their role and broaden their potential clinical use.

Although the meta-analysis indicated that andrographolide derivatives can improve clinical symptoms, pulmonary function, and inflammatory status in patients with AECOPD, the core molecular targets and pharmacological mechanisms underlying these effects remain unclear. Given the limited database information on individual derivatives, we selected the parent compound AD for mechanistic prediction. The obtained results only serve as hypothetical mechanisms for derivative injections.

Network pharmacology identified 39 overlapping targets. Protein–protein interaction analysis suggested that TNF, IL-1β, NLRP3, and caspase-1 may represent important targets through which AD and its derivatives act in AECOPD. During exacerbations, TNF-α promotes neutrophil recruitment, matrix metalloproteinase release, and NF-κB activation, processes associated with emphysema and airway injury (Thomson et al., 2012; Salimi Asl et al., 2019). IL-1β is another major driver of airway and systemic inflammation in COPD. It promotes neutrophil recruitment and IL-8/matrix metalloproteinase responses and is elevated in sputum or plasma during exacerbations (Matarazzo et al., 2022). Activation of the NLRP3 inflammasome is associated with pyroptosis and apoptosis and is considered closely involved in both COPD pathogenesis and exacerbation (Markelić et al., 2022). In preclinical studies, NLRP3 knockdown or pharmacological inhibition reduced neutrophilic pulmonary inflammation (Wu et al., 2022; Wang et al., 2021). Reduced sirtuin 1 (SIRT1) expression in COPD may aggravate oxidative stress and epithelial senescence and promote transforming growth factor beta (TGF-β)/SMAD-driven epithelial–mesenchymal transition and small-airway fibrosis (Guan et al., 2020; Chang et al., 2024). Persistent activation of TGF-β/SMAD signaling can also drive airway remodeling in COPD (Brake et al., 2023). The coordinated modulation of these targets by AD may therefore address several interconnected components of AECOPD pathophysiology. Notably, all of these targets are closely related to inflammatory responses, providing mechanistic support for the improvements in inflammatory indicators observed with andrographolide-derivative injections in the meta-analysis.

Functional enrichment analyses further supported this mechanistic hypothesis. GO and KEGG analyses suggested that AD may regulate fundamental pathological processes in AECOPD. Significant enrichment of cellular responses to chemical stimuli and apoptotic processes is consistent with mechanisms involved in maintaining the airway epithelial barrier and regulating epithelial-cell turnover. KEGG analysis further identified the NOD-like receptor and TNF signaling pathways as potential mechanistic hubs. COPD is closely associated with NOD-like receptor signaling: NOD1 and NOD2 may participate in innate airway sensing, whereas NLRP3 is among the best-supported downstream effectors of COPD-related inflammation (Gungor et al., 2025). TNF signaling is also strongly implicated in COPD. Through TNF receptor 1, TNF-α activates NF-κB and mitogen-activated protein kinase signaling, thereby promoting inflammation, mucus secretion, airway remodeling, and acute exacerbations (Budroni et al., 2024; Jiang et al., 2025). Both signaling pathways may exacerbate inflammatory responses in AECOPD through the recruitment of immune cells, promotion of pro-inflammatory cytokine production, and impairment of mucosal barrier integrity. Furthermore, NLRP3, caspase-1, and IL-1β, identified as pivotal targets in the PPI network, constitute key components of the NOD-like receptor signaling pathway. We therefore hypothesized that AD and its derivatives may act, at least in part, by modulating NLRP3-mediated inflammatory signaling.

To further verify the results obtained from network pharmacology, molecular docking was conducted to investigate the binding potential of AD with key target proteins (Yan et al., 2025). AD was docked with the principal proteins of the NLRP3 inflammatory pathway, including NLRP3, caspase-1, IL-1β, and IL-18. Molecular docking suggested energetically favorable predicted interactions between AD and NLRP3, caspase-1, IL-1β, and IL-18. These computational findings are consistent with the targets identified by network pharmacology and provide additional support for the proposed involvement of NLRP3-associated inflammatory signaling. However, docking results represent *in silico* predictions rather than direct evidence of physical binding or target modulation, and the predicted compound–target interactions require further experimental validation.

Previous *in vivo* studies have shown that AD can regulate NLRP3 inflammasome expression; however, its direct anti-inflammatory effects in airway epithelial cells have not been fully established. In a mouse model induced by aerosolized elastase and lipopolysaccharide, AD regulated NLRP3 and caspase-1 protein expression and effectively attenuated pulmonary inflammation (Yu et al., 2024b). Another study reported that AD modulated NLRP3 levels in cigarette-smoke-extract-exposed mice through c-Jun N-terminal kinase activation (Zhang et al., 2026). These findings further support the predictions generated by our network pharmacology analysis. Nevertheless, the previous studies did not focus on airway epithelial cells and therefore did not clarify the cell-specific regulatory effects of AD. NLRP3 inflammasome-mediated inflammation is known to cause airway epithelial injury and even pyroptotic cell death (Xiao et al., 2018; Zhuang et al., 2026). To further elucidate the molecular mechanisms by which AD and its derivatives may improve AECOPD, we conducted *in vitro* experiments to determine whether AD regulates NLRP3 and caspase-1 protein expression and suppresses inflammation in pulmonary epithelial cells under AECOPD-like conditions. The *in vitro* experiments demonstrated that AD markedly attenuated lipopolysaccharide-induced damage in BEAS-2B cells, enhanced cell viability, and reduced inflammatory responses in airway epithelial cells. These protective effects were accompanied by modulation of NLRP3 inflammasome- and caspase-1-associated protein expression, as well as decreased production of IL-1β and TNF-α. Collectively, these observations are in agreement with previous evidence and indicate that NLRP3, caspase-1, and IL-1β may contribute to the therapeutic actions of AD and its derivatives in AECOPD. However, the detailed causal mechanisms require further experimental investigation.

## 5 Conclusion

This integrated study suggests that andrographolide derivative injections may be effective adjunctive therapies for AECOPD. Nevertheless, the clinical findings should be interpreted cautiously because of methodological limitations, substantial heterogeneity for several outcomes, and the restricted geographic setting of the included trials.

The results of network pharmacology, molecular docking, and *in vitro* studies have generated a focused mechanistic hypothesis involving the NLRP3/caspase-1/IL-1β inflammatory axis. These findings provide preliminary biological plausibility for the clinical observations; however, the present study did not specifically investigate individual andrographolide derivatives. Future work should integrate rigorously designed clinical trials, comparative pharmacological studies of individual derivatives, and targeted experiments that directly validate the proposed compound-target interactions and signaling pathways.

## Data Availability

The data supporting the conclusions of this article are included within the article and its Supplementary Material. Further inquiries can be directed to the corresponding author.
