# Supplementary material for: Efficacy and mechanisms of andrographolide derivative in acute exacerbations of chronic obstructive pulmonary disease: a meta-analysis, network pharmacology, molecular docking, and in vitro validation study
Source: Front Pharmacol. 2026 Sep 23;17:1915339. doi: 10.3389/fphar.2026.1915339 (PMC13642008; doi:10.3389/fphar.2026.1915339)
Supplement: Supplementary file 1 [file Supplementaryfile1.docx]

**Supplementary File**

Supplementary Table 1. Search strategies for all databases.

Pubmed

Search: ((((((((((Chronic Obstructive Pulmonary Diseases) OR (COPD)) OR (Chronic Obstructive Lung Disease)) OR (COAD)) OR (Chronic Obstructive Airway Disease)) OR (Airflow Obstruction, Chronic)) OR (Airflow Obstructions, Chronic)) OR (Chronic Airflow Obstructions)) OR (Chronic Airflow Obstruction)) OR ((((((((((Chronic Obstructive Pulmonary Diseases) OR (COPD)) OR (Chronic Obstructive Lung Disease)) OR (Chronic Obstructive Pulmonary Disease)) OR (COAD)) OR (Chronic Obstructive Airway Disease)) OR (Airflow Obstruction, Chronic)) OR (Airflow Obstructions, Chronic)) OR (Chronic Airflow Obstructions)) OR (Chronic Airflow Obstruction))) AND ((((((Andrographolide) OR (Andrographolide injection)) OR (Xiyanping injection)) OR (Chuanhuning injection)) OR (Yanhuning injection)) OR (Lianbizhi injection))

("pulmonary disease, chronic obstructive"[MeSH Terms] OR ("pulmonary"[All Fields] AND "disease"[All Fields] AND "chronic"[All Fields] AND "obstructive"[All Fields]) OR "chronic obstructive pulmonary disease"[All Fields] OR ("chronic"[All Fields] AND "obstructive"[All Fields] AND "pulmonary"[All Fields] AND "diseases"[All Fields]) OR "chronic obstructive pulmonary diseases"[All Fields] OR ("pulmonary disease, chronic obstructive"[MeSH Terms] OR ("pulmonary"[All Fields] AND "disease"[All Fields] AND "chronic"[All Fields] AND "obstructive"[All Fields]) OR "chronic obstructive pulmonary disease"[All Fields] OR "copd"[All Fields]) OR ("pulmonary disease, chronic obstructive"[MeSH Terms] OR ("pulmonary"[All Fields] AND "disease"[All Fields] AND "chronic"[All Fields] AND "obstructive"[All Fields]) OR "chronic obstructive pulmonary disease"[All Fields] OR ("chronic"[All Fields] AND "obstructive"[All Fields] AND "lung"[All Fields] AND "disease"[All Fields]) OR "chronic obstructive lung disease"[All Fields]) OR ("pulmonary disease, chronic obstructive"[MeSH Terms] OR ("pulmonary"[All Fields] AND "disease"[All Fields] AND "chronic"[All Fields] AND "obstructive"[All Fields]) OR "chronic obstructive pulmonary disease"[All Fields] OR "coad"[All Fields]) OR ("pulmonary disease, chronic obstructive"[MeSH Terms] OR ("pulmonary"[All Fields] AND "disease"[All Fields] AND "chronic"[All Fields] AND "obstructive"[All Fields]) OR "chronic obstructive pulmonary disease"[All Fields] OR ("chronic"[All Fields] AND "obstructive"[All Fields] AND "airway"[All Fields] AND "disease"[All Fields]) OR "chronic obstructive airway disease"[All Fields]) OR ("pulmonary disease, chronic obstructive"[MeSH Terms] OR ("pulmonary"[All Fields] AND "disease"[All Fields] AND "chronic"[All Fields] AND "obstructive"[All Fields]) OR "chronic obstructive pulmonary disease"[All Fields] OR ("airflow"[All Fields] AND "obstruction"[All Fields] AND "chronic"[All Fields]) OR "airflow obstruction chronic"[All Fields]) OR ("pulmonary disease, chronic obstructive"[MeSH Terms] OR ("pulmonary"[All Fields] AND "disease"[All Fields] AND "chronic"[All Fields] AND "obstructive"[All Fields]) OR "chronic obstructive pulmonary disease"[All Fields] OR ("airflow"[All Fields] AND "obstructions"[All Fields] AND "chronic"[All Fields]) OR "airflow obstructions chronic"[All Fields]) OR ("pulmonary disease, chronic obstructive"[MeSH Terms] OR ("pulmonary"[All Fields] AND "disease"[All Fields] AND "chronic"[All Fields] AND "obstructive"[All Fields]) OR "chronic obstructive pulmonary disease"[All Fields] OR ("chronic"[All Fields] AND "airflow"[All Fields] AND "obstructions"[All Fields]) OR "chronic airflow obstructions"[All Fields]) OR ("pulmonary disease, chronic obstructive"[MeSH Terms] OR ("pulmonary"[All Fields] AND "disease"[All Fields] AND "chronic"[All Fields] AND "obstructive"[All Fields]) OR "chronic obstructive pulmonary disease"[All Fields] OR ("chronic"[All Fields] AND "airflow"[All Fields] AND "obstruction"[All Fields]) OR "chronic airflow obstruction"[All Fields]) OR ("pulmonary disease, chronic obstructive"[MeSH Terms] OR ("pulmonary"[All Fields] AND "disease"[All Fields] AND "chronic"[All Fields] AND "obstructive"[All Fields]) OR "chronic obstructive pulmonary disease"[All Fields] OR ("chronic"[All Fields] AND "obstructive"[All Fields] AND "pulmonary"[All Fields] AND "diseases"[All Fields]) OR "chronic obstructive pulmonary diseases"[All Fields] OR ("pulmonary disease, chronic obstructive"[MeSH Terms] OR ("pulmonary"[All Fields] AND "disease"[All Fields] AND "chronic"[All Fields] AND "obstructive"[All Fields]) OR "chronic obstructive pulmonary disease"[All Fields] OR "copd"[All Fields]) OR ("pulmonary disease, chronic obstructive"[MeSH Terms] OR ("pulmonary"[All Fields] AND "disease"[All Fields] AND "chronic"[All Fields] AND "obstructive"[All Fields]) OR "chronic obstructive pulmonary disease"[All Fields] OR ("chronic"[All Fields] AND "obstructive"[All Fields] AND "lung"[All Fields] AND "disease"[All Fields]) OR "chronic obstructive lung disease"[All Fields]) OR ("pulmonary disease, chronic obstructive"[MeSH Terms] OR ("pulmonary"[All Fields] AND "disease"[All Fields] AND "chronic"[All Fields] AND "obstructive"[All Fields]) OR "chronic obstructive pulmonary disease"[All Fields] OR ("chronic"[All Fields] AND "obstructive"[All Fields] AND "pulmonary"[All Fields] AND "disease"[All Fields])) OR ("pulmonary disease, chronic obstructive"[MeSH Terms] OR ("pulmonary"[All Fields] AND "disease"[All Fields] AND "chronic"[All Fields] AND "obstructive"[All Fields]) OR "chronic obstructive pulmonary disease"[All Fields] OR "coad"[All Fields]) OR ("pulmonary disease, chronic obstructive"[MeSH Terms] OR ("pulmonary"[All Fields] AND "disease"[All Fields] AND "chronic"[All Fields] AND "obstructive"[All Fields]) OR "chronic obstructive pulmonary disease"[All Fields] OR ("chronic"[All Fields] AND "obstructive"[All Fields] AND "airway"[All Fields] AND "disease"[All Fields]) OR "chronic obstructive airway disease"[All Fields]) OR ("pulmonary disease, chronic obstructive"[MeSH Terms] OR ("pulmonary"[All Fields] AND "disease"[All Fields] AND "chronic"[All Fields] AND "obstructive"[All Fields]) OR "chronic obstructive pulmonary disease"[All Fields] OR ("airflow"[All Fields] AND "obstruction"[All Fields] AND "chronic"[All Fields]) OR "airflow obstruction chronic"[All Fields]) OR ("pulmonary disease, chronic obstructive"[MeSH Terms] OR ("pulmonary"[All Fields] AND "disease"[All Fields] AND "chronic"[All Fields] AND "obstructive"[All Fields]) OR "chronic obstructive pulmonary disease"[All Fields] OR ("airflow"[All Fields] AND "obstructions"[All Fields] AND "chronic"[All Fields]) OR "airflow obstructions chronic"[All Fields]) OR ("pulmonary disease, chronic obstructive"[MeSH Terms] OR ("pulmonary"[All Fields] AND "disease"[All Fields] AND "chronic"[All Fields] AND "obstructive"[All Fields]) OR "chronic obstructive pulmonary disease"[All Fields] OR ("chronic"[All Fields] AND "airflow"[All Fields] AND "obstructions"[All Fields]) OR "chronic airflow obstructions"[All Fields]) OR ("pulmonary disease, chronic obstructive"[MeSH Terms] OR ("pulmonary"[All Fields] AND "disease"[All Fields] AND "chronic"[All Fields] AND "obstructive"[All Fields]) OR "chronic obstructive pulmonary disease"[All Fields] OR ("chronic"[All Fields] AND "airflow"[All Fields] AND "obstruction"[All Fields]) OR "chronic airflow obstruction"[All Fields]))) AND ("andrographolide"[Supplementary Concept] OR "andrographolide"[All Fields] OR "andrographolides"[All Fields] OR (("andrographolide"[Supplementary Concept] OR "andrographolide"[All Fields] OR "andrographolides"[All Fields]) AND ("inject"[All Fields] OR "injectability"[All Fields] OR "injectant"[All Fields] OR "injectants"[All Fields] OR "injectate"[All Fields] OR "injectates"[All Fields] OR "injected"[All Fields] OR "injectible"[All Fields] OR "injectibles"[All Fields] OR "injecting"[All Fields] OR "injections"[MeSH Terms] OR "injections"[All Fields] OR "injectable"[All Fields] OR "injectables"[All Fields] OR "injection"[All Fields] OR "injects"[All Fields])) OR ("Xiyanping"[All Fields] AND ("inject"[All Fields] OR "injectability"[All Fields] OR "injectant"[All Fields] OR "injectants"[All Fields] OR "injectate"[All Fields] OR "injectates"[All Fields] OR "injected"[All Fields] OR "injectible"[All Fields] OR "injectibles"[All Fields] OR "injecting"[All Fields] OR "injections"[MeSH Terms] OR "injections"[All Fields] OR "injectable"[All Fields] OR "injectables"[All Fields] OR "injection"[All Fields] OR "injects"[All Fields])) OR ("Chuanhuning"[All Fields] AND ("inject"[All Fields] OR "injectability"[All Fields] OR "injectant"[All Fields] OR "injectants"[All Fields] OR "injectate"[All Fields] OR "injectates"[All Fields] OR "injected"[All Fields] OR "injectible"[All Fields] OR "injectibles"[All Fields] OR "injecting"[All Fields] OR "injections"[MeSH Terms] OR "injections"[All Fields] OR "injectable"[All Fields] OR "injectables"[All Fields] OR "injection"[All Fields] OR "injects"[All Fields])) OR (("yanhuning"[Supplementary Concept] OR "yanhuning"[All Fields]) AND ("inject"[All Fields] OR "injectability"[All Fields] OR "injectant"[All Fields] OR "injectants"[All Fields] OR "injectate"[All Fields] OR "injectates"[All Fields] OR "injected"[All Fields] OR "injectible"[All Fields] OR "injectibles"[All Fields] OR "injecting"[All Fields] OR "injections"[MeSH Terms] OR "injections"[All Fields] OR "injectable"[All Fields] OR "injectables"[All Fields] OR "injection"[All Fields] OR "injects"[All Fields])) OR ("Lianbizhi"[All Fields] AND ("inject"[All Fields] OR "injectability"[All Fields] OR "injectant"[All Fields] OR "injectants"[All Fields] OR "injectate"[All Fields] OR "injectates"[All Fields] OR "injected"[All Fields] OR "injectible"[All Fields] OR "injectibles"[All Fields] OR "injecting"[All Fields] OR "injections"[MeSH Terms] OR "injections"[All Fields] OR "injectable"[All Fields] OR "injectables"[All Fields] OR "injection"[All Fields] OR "injects"[All Fields])))

WOS-

Chronic Obstructive Pulmonary Diseases OR COPD OR Chronic Obstructive Lung Disease OR Chronic Obstructive Pulmonary Disease OR COAD OR Chronic Obstructive Airway Disease OR Airflow Obstruction, Chronic OR Airflow Obstructions, Chronic OR Chronic Airflow Obstructions OR Chronic Airflow Obstruction (All Fields) and Andrographolide OR Andrographolide injection OR xiyanping injection OR chuanhuning injection OR yanhuning injection OR lianbizhi injection (All Fields) Embase-

'andrographolide derivatives' OR 'andrographolide injection' OR 'xiyanping injection' OR 'chuanhuning injection' OR 'yanhuning injection' OR 'lianbizhi injection' AND 'Chronic Obstructive Pulmonary Diseases' OR 'COPD' OR 'Chronic Obstructive Lung Disease' OR 'Chronic Obstructive Pulmonary Disease' OR “COAD” OR 'Chronic Obstructive Airway Disease' OR 'Airflow Obstruction, Chronic' OR 'Airflow Obstructions, Chronic' OR 'Chronic Airflow Obstructions' OR 'chronic obstructive lung disease'

Cochrane library-

'andrographolide derivatives' OR 'andrographolide injection' OR 'xiyanping injection' OR 'chuanhuning injection' OR 'yanhuning injection' OR 'lianbizhi injection' in All Text AND 'Chronic Obstructive Pulmonary Diseases' OR 'COPD' OR 'Chronic Obstructive Lung Disease' OR 'Chronic Obstructive Pulmonary Disease' OR “COAD” OR 'Chronic Obstructive Airway Disease' OR 'Airflow Obstruction, Chronic' OR 'Airflow Obstructions, Chronic' OR 'Chronic Airflow Obstructions' OR 'chronic obstructive lung disease' in All Text - (Word variations have been searched)

CNKI

(主题：慢阻肺急性加重+AECOPD+慢性阻塞性肺疾病急性加重+慢性阻塞性肺疾病急性发作+copd+慢阻肺)AND(主题：穿心莲+穿心莲内酯+喜炎平注射液+穿琥宁注射液+莲必治注射液+炎琥宁注射液)

Wanfang

主题:(慢阻肺急性加重 or AECOPD or 慢性阻塞性肺疾病急性加重 or 慢性阻塞性肺疾病急性发作or copd or 慢阻肺) and 主题:(穿心莲 or 穿心莲内酯 or 喜炎平注射液 or 穿琥宁注射液 or 莲必治注射液 or 炎琥宁注射液)

VIP-29

题名或关键词=慢阻肺急性加重 or AECOPD or慢性阻塞性肺疾病急性加重or慢性阻塞性肺疾病急性发作 or copd or 慢阻肺 AND 题名或关键词=穿心莲or穿心莲内酯or喜炎平注射液or穿琥宁注射液or莲必治注射液or 炎琥宁注射液

Supplementary Table 2：GRADE certainty of evidence assessment for main outcomes.

| **Outcome** | **Participants (Studies)** | **Risk of Bias** | **Inconsistency** | **Indirectness** | **Imprecision** | **Publication Bias** | **Certainty** |
| --- | --- | --- | --- | --- | --- | --- | --- |
| Clinical efficacy | 1922 | Serious | Not serious | Not serious | Not serious | Undetected | Moderate |
| FEV1 | 714 | Serious | Not serious | Not serious | Not serious | Undetected | Moderate |
| FEV1% | 806 | Serious | Not serious | Not serious | Not serious | Undetected | Moderate |
| PaO₂ | 344 | Serious | Not serious | Not serious | Not serious | Undetected | Moderate |
| CRP | 570 | Serious | Serious | Not serious | Not serious | Undetected | Low |
| WBC | 524 | Serious | Serious | Not serious | Not serious | Undetected | Low |
| NEUT% | 426 | Serious | Serious | Not serious | Not serious | Undetected | Low |
| PCT | 333 | Serious | Not serious | Not serious | Serious | Undetected | Low |

Supplementary Table 3：Subgroup analyses of treatment effects according to predefined factors.

Effective rate

| Category | Subgroup1 | Subgroup2 | Between-group difference |
| --- | --- | --- | --- |
| Dosage form | 1.22[1.16,1.27] | 1.20[1.10,1.31] | P=0.78 |
| Age | 1.19[1.13,1.26] | 1.24[1.16,1.32] | P=0.41 |
| Treatment course | 2.97[2.08,4.32] | 3.97[2.69,5.85] | P=0.28 |

PaO_2_

| Category | Subgroup1 | Subgroup2 | Between-group difference |
| --- | --- | --- | --- |
| Dosage form | 4.47[3.26, 5.29] | 6.68[4.83, 8.53] | P=0.03 |
| Age | 6.30[3.95, 8.64] | 6.18[4.77, 7.59] | P=0.93 |
| Treatment course | 4.48[2.47, 6.49] | 4.67[3.84, 5.51] | P=0.86 |

FEV1%

| Category | Subgroup1 | Subgroup2 | Between-group difference |
| --- | --- | --- | --- |
| Dosage form | 5.97[2.85, 9.10] | 9.66[-5.04, 24.36] | P=0.63 |
| Age | 6.37[1.81, 10.93] | 6.61[1.87, 11.36] | P=0.94 |
| Treatment course | 5.97[0.61, 11.34] | 7.45[2.82, 12.09] | P=0.68 |

FEV1

| Category | Subgroup1 | Subgroup2 | Between-group difference |
| --- | --- | --- | --- |
| Dosage form | 0.27[0.07, 0.47] | 0.32[0.21, 0.43] | P=0.68 |
| Age | 0.18[0.12, 0.23] | 0.36[0.22,0.49] | P=0.02 |
| Treatment course | 0.24[0.15, 0.33] | 0.41[0.09, 0.73] | P=0.33 |

FVC

| Category | Subgroup1 | Subgroup2 | Between-group difference |
| --- | --- | --- | --- |
| Dosage form | 0.25[0.03, 0.48] | 0.29[0.01, 0.57] | P=0.84 |
| Treatment course | 0.23[0.05, 0.41] | 0.32[-0.01, 0.64] | P=0.68 |

CRP

| Category | Subgroup1 | Subgroup2 | Between-group difference |
| --- | --- | --- | --- |
| Age | 6.25[3.52, 8.98] | 2.45[0.98, 3.91] | P=0.02 |
| Treatment course | 5.00[2.98,7.02] | 2.78[1.19,4.36] | P=0.04 |

WBC

| Category | Subgroup1 | Subgroup2 | Between-group difference |
| --- | --- | --- | --- |
| Age | 1.02[0.32,1.73] | 1.79[0.96,2.63] | P=0.17 |

NEUT%

| Category | Subgroup1 | Subgroup2 | Between-group difference |
| --- | --- | --- | --- |
| Age | 4.75[3.44,6.06] | 5.51[3.57,7.44] | P=0.52 |

PCT

| Category | Subgroup1 | Subgroup2 | Between-group difference |
| --- | --- | --- | --- |
| Dosage form | 0.15[0.06,0.25] | 0.18[0.09,0.27] | P=0.67 |
| Treatment course | 0.16[0.09,0.23] | 0.24[-0.01,0.49] | P=0.55 |

Supplementary figure1 Risk of bias summary

Supplementary Figure 2. Funnel plot, Egger's plot and trim-and-fill for overall response rate

Supplementary figure 3. Funnel plot and Egger's plot for PaO_2_

Supplementary figure 4. Funnel plot and Egger's plot for FEV_1_%

Supplementary figure5 Funnel plot and Egger's plot for FEV_1_

Supplementary Figure 6. Funnel plot and Egger's plot for CRP

Supplementary Figure 7. Uncropped Western Blot Images of NLRP3

Supplementary Figure 8. Uncropped Western Blot Images of caspase-1
